# Transcriptomic and chromatin accessibility profiling unveils new regulators of heat hormesis in *Caenorhabditis elegans*

**DOI:** 10.1371/journal.pbio.3003639

**Published:** 2026-02-20

**Authors:** Hsin-Yun Chang, Sarah E. McMurry, Sicheng Ma, Charles L. Heinke, Christian A. Mansour, Sophia Marie T. Schwab, Charles G. Danko, Siu Sylvia Lee

**Affiliations:** 1 Department of Molecular Biology and Genetics, Cornell University, Ithaca, New York, United States of America; 2 Department of Biomedical Science, Cornell University, Ithaca, New York, United States of America; Korea Advanced Institute of Science and Technology, KOREA, REPUBLIC OF

## Abstract

Heat hormesis describes the beneficial adaptations resulting from transient exposure to mild heat stress, which enhances stress resilience and promotes healthy aging. While heat hormesis is widely observed, much remains to be learned about its molecular basis. This study bridges a critical knowledge gap through a comprehensive multiomic analysis, providing key insights into the transcriptomic and chromatin accessibility landscapes throughout a heat hormesis regimen in *Caenorhabditis elegans*. We uncover highly dynamic, dose-dependent molecular responses to heat stress and reveal that while most initial molecular changes induced by mild stress revert to baseline, key differences emerge in response to subsequent heat shock challenge that likely contribute to physiological benefits. We further demonstrate that heat hormesis extends life span specifically in wild-type animals, but not in germline-less mutants, likely due to transient disruption of germline activities during mild heat exposure, which appears sufficient to trigger pro-longevity mechanisms. This finding points to tissue-specific responses in mediating the physiological outcomes of heat hormesis. Importantly, we identify several highly conserved regulators of heat hormesis that likely orchestrate gene expression to enhance stress resilience. Among these regulators, some (MARS-1/MARS1, SNPC-4/SNAPc, FOS-1/c-Fos) are broadly required for heat-hormesis-induced benefits, whereas others (ELT-2/GATA4, DPY-27/SMC4) are uniquely important in specific genetic backgrounds. This study advances our understanding of stress resilience mechanisms, points to multiple new avenues for future investigations, and provides a molecular framework for promoting healthy aging through strategic mid-life stress management.

## Abstract 2

Exposure to mild heat stress can induce adaptations that promote stress resilience and healthy aging, in a process termed heat hormesis. This study provides a detailed multiomic characterization of the transcriptomic and chromatin accessibility changes that occur during heat hormesis in C elegans, and identifies several novel regulators of this process.

## Introduction

Transient exposure to mild stress is well known to activate adaptive mechanisms that confer long-term protective effects and promote health, a phenomenon known as hormesis [1–4]. Specifically, ‘heat hormesis’ describes the beneficial outcomes triggered by mild thermal stress, documented across species from yeast to humans [4–13]. Sauna therapy, for instance, is increasingly recognized for its potential to improve health and mitigate age-related diseases through mechanisms attributed to heat hormesis [14,15]. Despite extensive observational evidence of the beneficial effects of heat hormesis across diverse species and growing popularity in wellness practices, the molecular mechanisms underlying heat hormesis remain incompletely understood. *Caenorhabditis elegans* represents an ideal model for dissecting heat hormesis mechanisms due to its genetic tractability, well-characterized stress responses, and short life span.

In *C. elegans,* exposure to elevated temperatures at various life stages, including short exposures in young adulthood [4,6,16–19] or chronic exposure throughout development [20,21], consistently boosts thermotolerance and extends life span. Genetic analyses reveal a critical role of the transcription factor HSF-1 in mediating heat hormesis [17,19,22–25]. HSF-1 is well-established to mount a robust transcriptional program in response to heat stress, which includes rapid induction of heat shock protein (hsp) genes. Additional stress response transcription factors, such as DAF-16/FOXO, HIF-1, HLH-30/TFEB, have also been implicated in heat hormesis [19,20,22–27]. Recent studies also highlight chromatin regulators, such as histone acetyltransferase CBP-1 and histone remodeler SWI/SNF, as participants in the long-term beneficial effects of heat hormesis [21,28].

Using transcriptomic profiling, a recent study identified distinct patterns of gene expression changes of *C. elegans* after a short exposure to heat stress (35 °C for 1 hour) and 4 hours after recovery at normal growth temperature, including genes whose expression persists throughout the 4 hours of recovery, as well as genes that continue to be induced during recovery [29]. Interestingly, some changes specific to the recovery period are regulated by the endoribonuclease ENDU-2, independent of HSF-1 [29]. Another transcriptomic study comparing *C. elegans* raised at 15 °C versus 25 °C (as a model of chronic stress exposure) revealed differential gene expression profiles between the two populations [21], indicating *C. elegans* exhibits specific gene expression patterns in response to different temperatures.

Despite these advances, a longitudinal multiomic analysis that tracks changes across the entire heat hormesis regimen, including subsequent stress challenge, remains unexplored. Such data could provide crucial molecular insights that correlate with the improved physiological outcomes observed under hormesis conditions and identify possible regulators of the protective effects. This study fills this gap by presenting detailed transcriptomic and chromatin accessibility profiles at key timepoints throughout a heat hormesis regimen. We find that mild heat exposure induces extensive changes in RNA expression and chromatin accessibility that, although largely restored after a recovery period, leave distinct molecular imprints upon subsequent heat shock (HS). Our findings illustrate the dynamic molecular landscape during heat hormesis, providing concrete molecular evidence for dose-dependent responses and uncovering new candidate regulators with critical roles in diverse biological functions. Notably, multiomic analyses in wild-type (WT) and the germline-less mutant *glp-1(ts)* point to transient disruption of germline activities during mild heat stress exposure, which contributes to longevity extension, indicating tissue-specific responses to heat stress can have lasting effects on organismal physiology. Furthermore, among the candidate regulators we uncovered, some show context-dependent regulation of heat hormesis, while others are broadly important. Our study offers valuable insights into the molecular basis for the development of stress resilience and may pave the way for promoting healthy aging through stress management.

## Results

### Priming induces thermotolerance

To understand the molecular basis of heat hormesis, we adapted a regimen in which *C. elegans* that had been cultured at 20 °C were exposed to 30 °C for 6 hours during early adulthood (referred to as “priming”), allowed to recover at 20 °C, and then challenged with HS at 35 or 37 °C. We applied this hormesis regimen to WT or *glp-1(e2144)* mutant (which become germline-less when grown at the non-permissive temperature of 25 °C, hereafter referred to as *glp-1(ts)*) worms) and tested their thermotolerance after HS.

**Fig 1:**
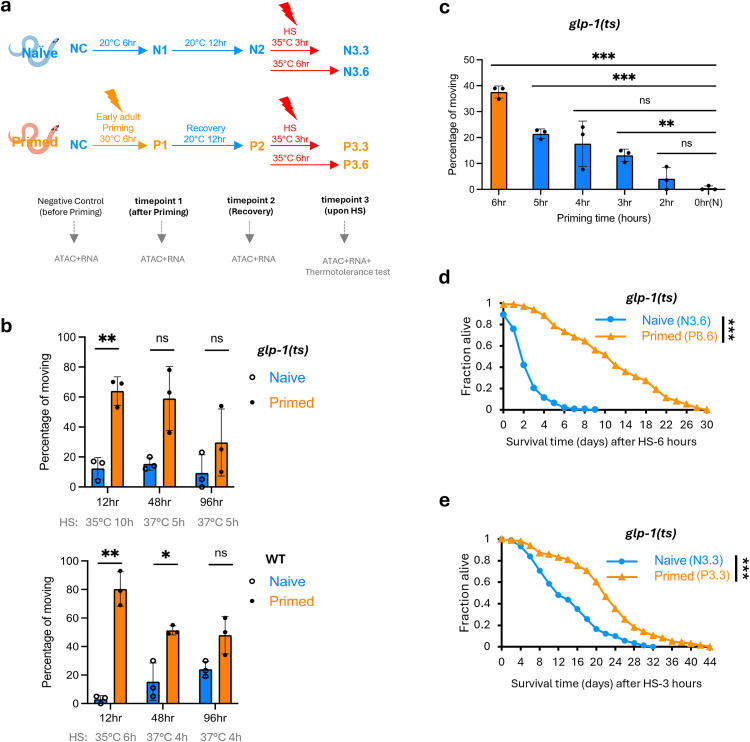
A heat hormesis regimen robustly enhances thermotolerance. **(a)** Schematic of heat hormesis regimen and timepoints of multiomics studies: The primed group was exposed to 30 °C for 6 hours (priming) during the early adult stage, followed by 12 hours of recovery at 20 °C. The naive group was incubated at 20 °C concurrently. Both groups were subjected to subsequent heat shock (HS) challenge at the indicated temperature and duration. Negative control (NC) time point was collected before priming. For both naive and primed groups, time point 1 (T1) was collected after priming (N1 and P1), time point 2 (T2) after recovery (N2 and P2), and timepoints 3 (T3) after 3 hours or 6 hours of HS (N3.3, P3.3, N3.6, P3.6). Created in BioRender. Lee, S. (2026) https://BioRender.com/uewxn69. **(b)** Thermotolerance of *glp-1(ts)* (*e2144* temperature-sensitive allele) (top) and WT (bottom), measured by the percentage of worms moving 15 h post-HS. The y-axis represents average results from three separate experiments, each with a different recovery period between priming and subsequent HS challenge. Recovery times and HS conditions varied due to the ease of experimental design. *glp-1(ts):*12 h recovery followed by 10 h of HS at 35 °C; 48 h recovery followed by 5 h of HS at 37 °C; 96 h of recovery followed by 5 h of HS at 37 °C (*N* = 3); WT: 12 h recovery followed by 6 h HS at 35 °C; 48 h recovery followed by 4 h HS at 37 °C; 96 h recovery followed by 4 h HS at 37 °C (*N* = 3). **(c)** Thermotolerance of *glp-1(ts)* worms underwent various priming durations at 30 °C, followed by 12 h recovery at 20 °C, prior to a 10-hour HS at 35 °C. Movement was scored 15 hours post-HS. Statistical analyses were conducted by comparing the indicated priming time to the 0-hour (Naive). (*N* = 3). (**d, e)** Survival of *glp-1(ts)* worms after HS 6 h (**d**) or 3 h (**e**) at 35 °C, following a 12 h recovery from priming. The figure represents combined data from three independent experiments (*N* = 3). A 2-tailed unequal variances *t* test was conducted to compare differences between naive and primed groups in **(b–e)**. ** Indicates *p* < 0.01, *** Indicates *p* < 0.001. Numerical data from independent replicates can be found in S1 Data.

We found that priming significantly improved thermotolerance, based on motility scoring, in WT and *glp-1(ts)* mutant worms. The enhanced thermotolerance persisted after 12- or 48-hours of recovery at 20 °C (Fig 1B), but the effect waned after a 96-hour recovery period. These results indicated that both WT and *glp-1(ts)* worms can retain a “memory” of the priming experience for an extended period, thereby exhibiting enhanced resistance to subsequent HS challenge. We evaluated various priming durations and determined that a 6-hour priming induced a greater protective effect compared to shorter priming times (Fig 1C). We additionally monitored survival after HS and observed that the primed worms lived longer than their naive counterparts post HS (Fig 1D and 1E).

### Transcriptomic and chromatin accessibility profiling in heat hormesis

To investigate the molecular basis of how transient exposure to mild heat stress, i.e., priming, confers resistance to a more intense HS later, we profiled mRNA expression and chromatin accessibility using RNA-seq and ATAC-seq, respectively, at key timepoints across our heat hormesis regimen. We hypothesized that RNA-seq and ATAC-seq data together would provide a more comprehensive view of the dynamics of gene expression regulation across the heat hormesis regimen. We initially conducted the multi-omic analyses using *glp-1(ts)* mutant, which lacks germline cells, thus enabling the assessment of molecular changes in somatic cells, and minimizing the possible confounding effects associated with extensive gene expression dynamics during early adulthood of reproductive worms. The timepoints we chose, including immediately after the 6-hour priming at 30 °C (time point 1), after the 12-hour recovery at 20 °C (time point 2), and after 3 or 6 hours of HS at 35 °C (time point 3) (Fig 1A), were guided by the dramatic phenotypic differences between primed and naive worms after HS (Fig 1D and 1E).

We first examined RNA expression and chromatin accessibility changes in the naive group. We detected minor changes at timepoints 1 and 2 (N1 versus NC, N2 versus N1) (S2A Fig), likely reflecting developmental progression at normal culturing temperature. HS induced dramatic changes both in RNA expression and in chromatin accessibility (N3 versus N2) (S2A Fig). These changes showed significant overlap with previously reported heat stress-induced gene expression profiles (S2E Fig) [29,30], despite differences in experimental setup, supporting the validity of our results.

### Priming-responsive changes largely restore after recovery

**Fig 2:**
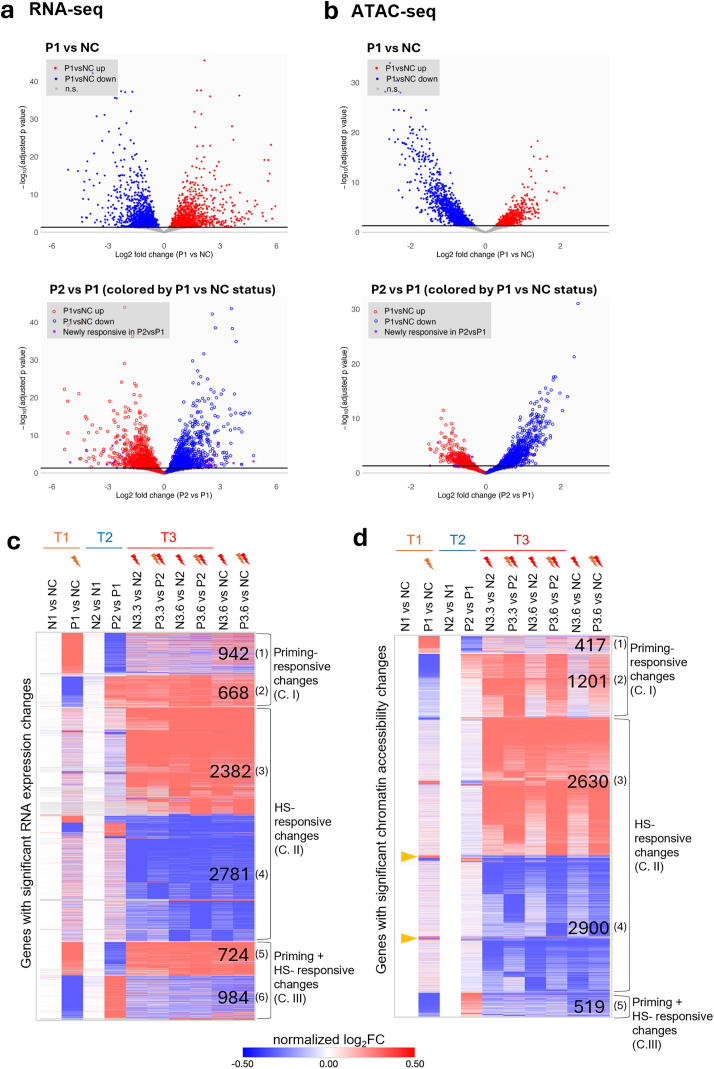
Priming-responsive changes largely restore after recovery and are distinct from heat shock-responsive changes in gene expression and chromatin accessibility. **(a)** RNA-seq and **(b)** ATAC-seq volcano plots. Top panels: Differential gene expression or chromatin accessibility after priming (P1 vs. NC). Red and blue indicate significantly up- or downregulated genes/peaks, respectively (adjusted p < 0.05, threshold shown as black horizontal line). Bottom panels: Differential gene expression or chromatin accessibility after recovery (P2 vs. P1), with genes/peaks colored by their P1 vs. NC status. Red and blue open circles represent genes/peaks previously up- or downregulated at P1 vs. NC, showing largely opposite regulation after recovery. Purple points indicate “newly responsive” genes/peaks that were not significantly changed in P1 vs. NC but became differentially regulated in P2 vs. P1. For RNA-seq **(a)**, the y-axis was truncated for visualization (Max = 45). Complete gene and peak lists are provided in S1 Data. Heatmaps display genes with significant changes in RNA expression **(c)** or genes linked to the regions with significant change in chromatin accessibility **(d)** across the indicated comparisons, clustered using k-means in Morpheus. Panel **(c)** includes 8,481 genes, and panel **(d)** includes 10,837 genes linked to 17,943 chromatin regions (peaks). Colors represent normalized log2FC. The color scale reflects a normalized range of log2FC, with the minimum and maximum log2FC values mapped to −0.5 and +0.5, respectively. This normalization does not imply that actual log2FC values are limited to this range; rather, the color mapping compresses the scale to visualize the full spectrum of fold changes within a standardized gradient. Numbers indicate the size of each cluster (genes in **c**; genes with associated peaks in **d**). The clusters were rearranged to highlight shared patterns between transcriptomic and chromatin accessibility changes. Heatmaps are organized into three categories: Category I (C. I), Priming-responsive changes, including clusters 1–2 in (c, d); Category II (C. II), HS–responsive changes, including clusters 3–4 in (c, d); and Category III (C. III), Priming + HS-responsive changes, including clusters 5–6 in (**c**) and cluster 5 in (d). A small subset of gene-associated peak regions (yellow arrowheads in **d**) gained accessibility upon priming but not during HS, more closely resembling the pattern of RNA-seq cluster 1. Details of the gene lists in the heatmaps can be found in S1 and S2 Data.

We next focused on the primed group: Both RNA-seq and ATAC-seq revealed substantial changes immediately after priming (P1 versus NC), with 1,390 genes and 808 peaks significantly upregulated and 1,423 genes and 2,110 peaks downregulated, respectively (Figs 2A and 2B, top and S2B). Strikingly, the vast majority of these priming-responsive changes reversed direction after a 12-hour recovery at 20 °C (P2 versus P1) (Fig 2A and 2B, bottom), indicating that the transcriptional and chromatin accessibility programs induced by priming are transient and reversible.

Upon HS at 35 °C, we observed dramatic changes in RNA expression and chromatin accessibility in the primed group (S2B Fig), similar to that in the naive group as discussed above (S2A Fig). We also compared the HS time point (T3.6) directly to baseline (NC), in addition to the time point after recovery. As expected, the two comparisons yielded nearly identical patterns (Fig 2C and 2D), supporting our conclusion that most priming-induced changes are transient and return to basal levels by time point 2.

A comparison between the RNA-seq and ATAC-seq results indicated that there was a significant overlap, as well as distinction, between the genes associated with RNA expression or chromatin accessibility changes through the hormesis regimen. The data together supported our notion that the two genomic assays together provided a more comprehensive view of the gene regulatory changes associated with our heat hormesis regimen.

### Different levels of heat stress induce largely distinct RNA or chromatin accessibility changes

To further characterize the dynamic changes in RNA expression and chromatin accessibility across our hormesis regimen, we performed K-means clustering analysis of the genes associated with significant changes in RNA-seq or ATAC-seq across the three timepoints in either the naive or primed groups, and the results were visualized using heatmaps. The heatmap of ‘Genes with significant RNA expression changes’ includes the total number of genes identified from RNA-seq data (Fig 2C), while the heatmap of “Genes with significant chromatin accessibility changes” includes the genes associated with the peaks identified from ATAC-seq data (Fig 2D). Interestingly, despite RNA-seq and ATAC-seq revealing substantially different genes associated with significant changes across the hormesis regimen, the clustering analyses revealed a similar pattern of changes (Fig 2C and 2D). We classified these shared patterns into three categories: Priming-responsive changes (C. I), HS-responsive changes (C. II), and Priming + HS-responsive changes (C. III) (Fig 2C and 2D).

Priming-responsive changes (C. I) describe those exhibiting either upregulation or downregulation after priming (P1 versus NC) and the opposite trend following recovery (P2 versus P1), again supporting our earlier conclusion that most of the priming-induced RNA and chromatin accessibility changes are restored after recovery. Changes in this category were not robustly recapitulated after HS (time point 3) (Fig 2C and 2D, Clusters (1) & (2)). The HS-responsive changes (C. II) displayed robust changes in RNA expression or chromatin accessibility upon HS (time point 3.3 or 3.6 versus time point 2), with minimal alterations observed after priming (time point 1) and following recovery (time point 2) (Fig 2C and 2D, Clusters (3) & (4)).

The final category (C. III) includes genes that responded to both priming and HS, with most exhibiting consistent up- or down-regulation upon priming and HS (Fig 2C and 2D, Clusters (5) & (6)). Interestingly, in this category, ATAC-seq data uncovered only genomic regions associated with significantly reduced chromatin accessibility upon priming and HS (Fig 2D). Close inspection revealed that many regions in ATAC-seq Cluster 1 showed a strong increase in accessibility after priming, but a weaker, yet still detectable, increase after HS (resembling the behavior of RNA-seq Cluster 5). We also observed a small set of regions that gained accessibility upon priming but not during HS (highlighted in Fig 2D), which more closely resemble RNA-seq Cluster 1. However, the limited number of these regions prevented them from forming a distinct cluster under our k-means approach.

To understand the potential biological relevance of the various changes, we conducted Gene Ontology (GO) analyses of the various clusters. For Priming-responsive changes (C. I), we note that “ribosome-related” and “stress response” were the functional groups associated with both higher and lower expression and more and less chromatin accessibility, perhaps reflecting the dynamic nature of these classes of genes during the priming period (S3A Fig). Similarly, for HS-responsive changes (C. II), “lipid metabolism” and “small GTPase signaling” were the functional groups represented by both more and less chromatin accessibility. Interestingly, “Transcription factor: NHR” was associated with both downregulated RNA expression and less open chromatin (S3B Fig). For the changes that are shared between priming and HS (C. III, Priming + HS-responsive changes), “mRNA processing,” “heat stress response,” and “proteolysis proteasome” were the top significantly enriched functional groups associated with genes with upregulated RNA expression (848). We note that canonical HS response genes are among this group, and their repeated induction likely contributes to the phenotypic protection conferred by heat hormesis. “Metabolism (lipid, short chain dehydrogenase)” and “detoxification stress response” were among the most significantly enriched GO terms for genes with downregulated RNA expression (980). Additionally, “metabolism (lipid and amino acid)” was significantly enriched for genes with less open chromatin (567) (S3C Fig). The downregulation of lipid metabolism genes may reflect a compensatory response to changes in membrane fluidity at high temperature. Details of the GO analysis can be found in S4 Data.

### Primed worms exhibit differential RNA expression and chromatin accessibility upon HS compared to naive worms

**Fig 3:**
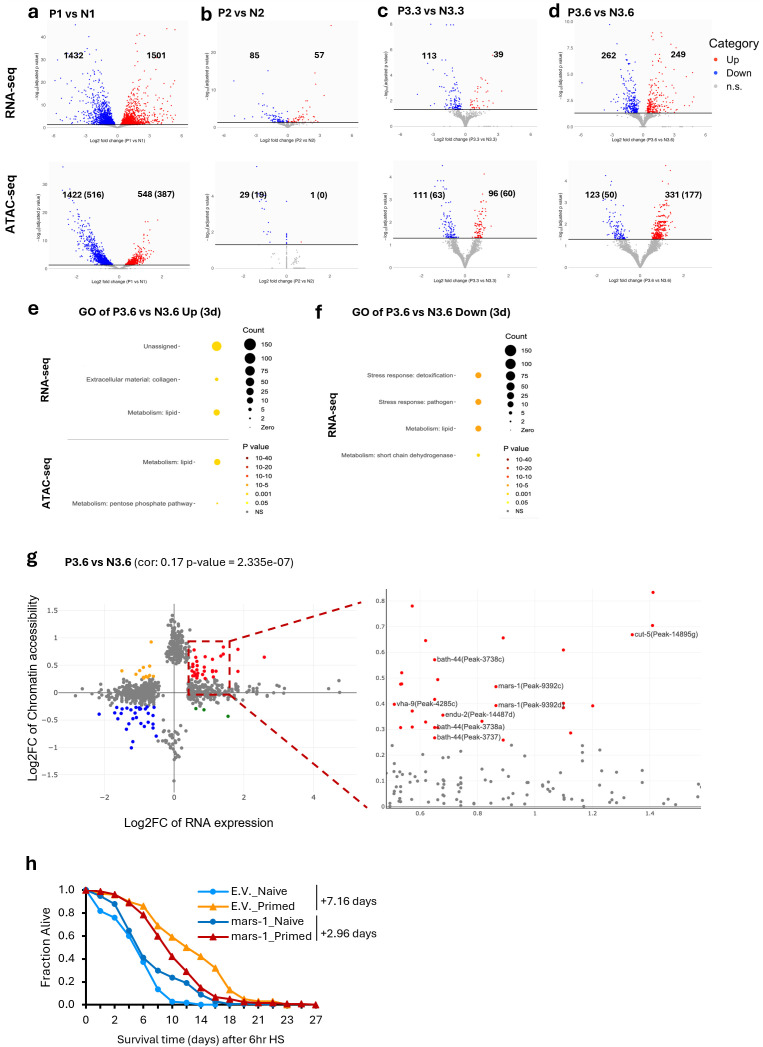
Differences in gene expression or chromatin accessibility in primed and naive worms likely contribute to the protective effects of heat hormesis. Volcano plots display differential gene expression (top panels) and chromatin accessibility (bottom panels) between primed and naive groups at the indicated timepoints: after priming **(a)**, after recovery **(b)**, after a 3-hour HS **(c)**, and after a 6-hour HS **(d)**. Red and blue indicate significantly up- and downregulated genes or peaks, respectively (adjusted p < 0.05; threshold shown as a black horizontal line). The numbers of significant genes or peaks (with associated genes in brackets) are indicated. Wormcat GO enrichment analysis for genes upregulated **(e)** or downregulated **(f)** in primed compared to naive groups after a 6-hour HS. Details are provided in S1 and S4 Data. **(g)** Scatter plot displays genes with significant changes identified in RNA expression or chromatin accessibility after a 6-hour HS, with correlated changes between RNA-seq and ATAC-seq data highlighted according to defined criteria: log2FC RNA expression > 0.5, < −0.5; log2FC Chromatin accessibility > 0.25, < −0.25. The area showing genes upregulated in both RNA expression and chromatin accessibility (red) is zoomed out in the right panel. The five candidate genes (and their associated peaks) selected for RNAi testing are labeled. **(h)** Thermotolerance was assessed based on the survival of *glp-1(ts)* worms treated with the indicated RNAi or empty vector (E.V.) after being subjected to our heat hormesis regimen and challenged with a 6-hour HS. Survival curves represent combined data from three independent experiments (*N* = 3). The survival extension day indicates the mean increase in survival time (in days) of the primed group relative to the naive group. Details can be found in S5 Data.

To uncover the molecular basis underlying the protective effects of primed worms, we assessed the differences in RNA expression and chromatin accessibility between primed and naive groups at the various timepoints. At time point 1, changes between primed and naive were largely similar to those detected when comparing P1 versus NC (Figs 3A and S2B). At time point 2, we detected only a handful of significant differences between primed and naive worms, which is consistent with our earlier conclusion that the majority of the priming-induced changes were restored after recovery (Fig 3B). Nevertheless, we identified a small subset of genes that exhibited significantly persistent RNA expression change through recovery (30 among 1,501 upregulated, 43 among 1,432 downregulated), including the HSP gene *hsp-12.3* (S4E and S4F Fig)*.*

Intriguingly, despite minimal differences observed between primed and naive worms post-recovery, significant differences in both RNA expression and chromatin accessibility were detected between the two groups after HS of 3 or 6 hours (Fig 3C and 3D), suggesting an underlying molecular “memory” of the earlier heat priming. As expected, the RNA expression and chromatin accessibility differences between primed and naive worms after 3 or 6 hours of HS showed a good degree of overlap (S4A Fig). Consistent with the phenotypic observation that primed worms showed greater survival advantage after a 6-hour HS compared to a 3-hour HS (Fig 1D and 1E), we detected substantially more RNA expression and chromatin accessibility differences between primed and naive worms after a 6-hour HS (Fig 3C and 3D). Specifically, 249 and 262 genes showed significantly up- or down-regulated RNA expression in the primed compared to the naive group (Fig 3D). Additionally, 331 peaks (associated with 177 genes) and 123 peaks (associated with 50 genes) were significantly up- or down-regulated in the primed group (Fig 3D). Among the genes with significantly upregulated expression (249) or more open chromatin (177) in the primed group, lipid metabolism and collagen were the significantly enriched GO term (Fig 3E); Among the genes with significantly downregulated expression (262), detoxification stress response, pathogen stress response, and lipid metabolism were among the significantly enriched GO terms (Fig 3F). We conducted additional clustering analysis to assess the temporal trajectories of RNA expression across our hormesis regimen between the primed and naive groups and came to a similar conclusion that most priming-induced gene expression changes are transient, but also highlighted different RNA expression dynamics between the primed and naive groups (S1 Text and S4C and S4D Fig). Since most of the RNA and chromatin accessibility changes are restored after recovery, future work examining the persistence of post-transcriptional and/or protein-level changes will likely be informative.

### WT exhibits patterns of RNA expression and chromatin accessibility dynamics across heat hormesis similar to those of germline-less mutant

To investigate whether our findings are broadly relevant, and not unique to the *glp-1(ts)* mutant, we performed a parallel multiomic analysis using WT N2 strain. WT worms showed transcriptomic and chromatin accessibility dynamics highly consistent with those observed in *glp-1(ts)* mutants across the heat hormesis regimen. In WT, we again observed that the priming-responsive changes were largely restored following recovery (S5A, S5B and S6B Figs), and that different levels of heat stress induced largely distinct transcriptomic and chromatin accessibility profiles (S5C and S5D Fig). Together, these results indicate that WT and *glp-1(ts)* share similar multiomic responses, supporting that some of the molecular changes underlying heat hormesis are broadly generalizable. Interestingly, we detected fewer differentially expressed genes in WT overall, consistent with the possibility that dynamic changes in the reproductive germline could obscure somatic responses to heat hormesis. Thus, while the WT data confirm the generality of our findings, the *glp-1(ts)* background provides a clearer readout of somatic regulation that confers thermotolerance and therefore serves as the primary dataset for identifying candidate regulators of heat hormesis.

We additionally applied the WormExp (V2.0) (https://wormexp.zoologie.uni-kiel.de/wormexp/) tool [33] to our priming-induced significantly changed genes (P1 versus N1) from *glp-1(ts)* and WT. This tool compares gene sets against a database that includes numerous gene expression profiles based on published studies in *C. elegans* and computes statistically significant enriched categories. Focusing on the top-ranked significantly enriched categories, we found that in *glp-1(ts)*, heat priming-induced upregulated genes were highly enriched for CSR-1–associated 22G-RNA targets and TGF-β-downregulated genes. These results suggest that changes in small RNA and TGF-β signaling pathways are a major molecular consequence of heat priming in germline-less animals. In WT, the categories included germline stem cell removal-associated genes and *glp-1(ts)*-upregulated genes (discussed further below). Additionally, gene sets associated with pathogen exposure were also highly enriched. Notably, in both WT and *glp-1(ts)* backgrounds, gene sets associated with the DAF-2/DAF-16-dependent IIS pathway and dietary restriction were also significantly enriched, although they were not among the top-ranked categories. Together, these findings suggest that while molecular responses to heat priming partially overlap with well-established longevity pathways like IIS, TGF-β signaling, and dietary restriction in both WT and *glp-1(ts)* backgrounds, they also appear to include unique regulatory programs (S1 Data: WormExp).

### Multiomic data unveil putative new regulators of thermotolerance induced by heat hormesis

To assess whether the differential molecular changes between primed and naive worms contributed to the protective effects induced by mild heat stress priming, we used two strategies to select candidate genes and tested their functional relevance in thermotolerance using the regimen described for Fig 1D.

The first strategy prioritized genes that showed overlapping upregulation in both RNA expression and chromatin accessibility between primed and naive worms after HS for 6 hours (P3.6 versus N3.6) (Fig 3G). Although some of the strongest overlapping hits (top right-most dots in Fig 3G) could not be tested due to a lack of available RNAi constructs in our library, we tested five candidates (*mars-1*, *endu-2*, *bath-44*, *vha-9*, *cut-5)* guided by their annotated gene functions. Among these, only *mars-1* knockdown significantly compromised the survival advantage of primed worms. *mars-1*, the ortholog of human MARS1, encodes a cytoplasmic methionyl-tRNA synthetase that catalyzes methionine attachment to its cognate tRNA, an essential step in protein synthesis. Specifically, priming induced a reduced survival benefit in worms treated with *mars-1* RNAi (mean survival extension of 2.96 days) compared to those treated with empty vector control (E.V.) (mean survival extension of 7.16 days, Fig 3H). Interestingly, in naive worms, *mars-1* knockdown results in increased survival after HS compared to control (Fig 3H). This is consistent with previous reports showing that *mars-1* RNAi enhances oxidative stress resistance and extends life span in *C. elegans* [31].

The second strategy focused on predicting the transcription factors that regulate the observed molecular changes, with the goal of identifying regulators that play a role in heat hormesis. We conducted motif analysis using significantly changed peak regions identified from ATAC-seq and the promoter regions of significantly changed genes from RNA-seq. The analysis revealed three groups of significantly enriched motifs: i) Motifs that were enriched based on both ATAC-seq and RNA-seq results; ii) Motifs enriched based on only RNA-seq or (iii) only ATAC-seq results (Table 1). From the list of enriched motifs (*Q* < 0.05) (S1 Data), we selected candidate factors that (1) were uncovered across the different datasets, (2) had available RNAi constructs, and (3) had prior evidence linking them to stress response or longevity, for further functional testing.

**Table 1:** Candidate transcription factors predicted by motif analysis and tested by RNAi.

Candidates	P1 vs. NC	P3.6 vs. P2	(P1 vs. NC) and (P3.6 vs. P2) intersect	P1 vs. N1	P3.6 vs. N3.6
cebp-1	RNA: up	RNA: up (2,000 bp)		RNA: up	
		ATAC: up	ATAC: up	ATAC: up	
**snpc-4**		RNA: up			
	ATAC: up			ATAC: up	
hlh-30	RNA: down (2,000 bp)	RNA: up (2,000 bp)			
	ATAC: down	ATAC: up			ATAC: up
**hsf-1 (CIS-BP)**		RNA: up	RNA: up		
		ATAC: up, down			
**elt-2**	RNA: down			RNA: down	RNA: down
elt-6	RNA: down			RNA: down	RNA: down
pqm-1	RNA: down			RNA: down	RNA: down
**dpy-27**	ATAC: down			ATAC: down	ATAC: down
**fos-1**		ATAC: up			
atf-7		ATAC: up			

This table shows the transcription factor candidates predicted by motif analysis that have been tested by RNAi, with at least two independent experiments. Promoter regions of genes associated with significant changes in RNA expression from RNA-seq and DNA regions spanning significantly changed peaks from ATAC-seq were subjected to motif analysis. Motif candidates were primarily selected from the JASPAR database, except for the HSF-1 motif, which is from CIS-BP database. Motifs identified from RNA-seq data were primarily enriched in the 500 bp upstream promoter region of the TSS, unless indicated as 2,000 bp. The complete list of all significantly enriched motifs (*Q* value < 0.05) is shown in S1 Data. The table is color-coded into three groups: Motifs enriched based on both ATAC-seq and RNA-seq results (pink); Motifs enriched based on only RNA-seq (green); Motifs enriched based on only ATAC-seq results (blue). In addition to HSF-1, three candidates from each group were selected for RNAi screening if they were repeatedly identified in different significant lists, RNAi constructs were available, and the candidate factor had been implicated in stress response and longevity according to the literature.

**Fig 4:**
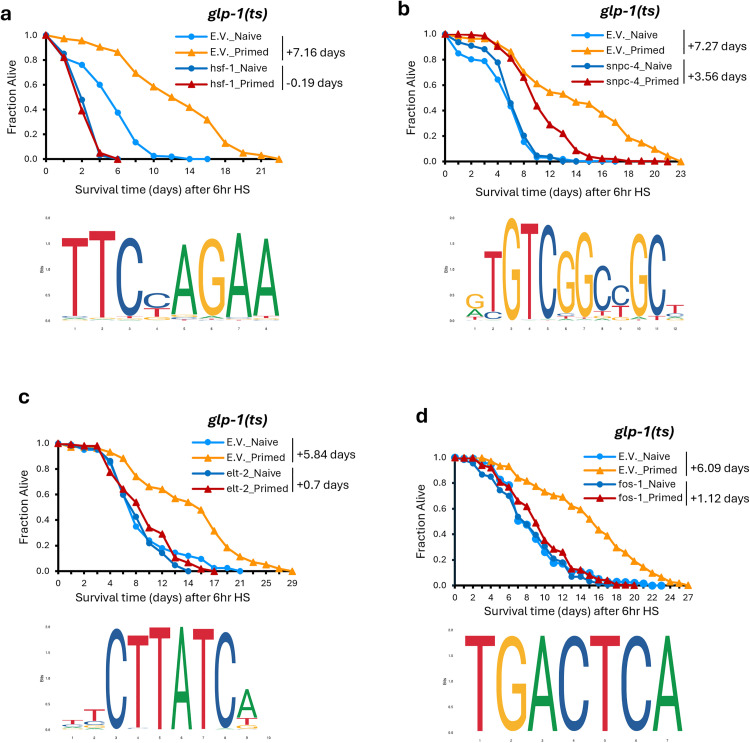
Motif analysis unveils regulators of heat hormesis. Thermotolerance was assessed based on survival of *glp-1(ts)* mutant worms treated with the indicated RNAi and after being subjected to our hormesis regimen and challenged with a 6-hour HS. Survival curves represent combined data from multiple independent experiments (N) for *glp-1*_Naive or *glp-1*_Primed treated with empty vector (E.V.) control RNAi or *hsf-1* RNAi **(a; *N* = 3)**, *snpc-4* RNAi **(b; *N* = 4)**, *elt-2* RNAi **(c; *N* = 2),** *fos-1* RNAi **(d; *N* = 4)**. RNAi targeting *hsf-1***(a)** and *elt-2* **(c)** completely abolished the enhanced survival to heat in primed groups. For the *snpc-4* **(b)** and *fos-1* **(d)** RNAi, which partially compromised the enhanced survival to heat in primed groups, the survival extension rate is calculated, and the increase in mean survival days in primed worms relative to naive worms is shown. Details can be found in S5 Data.

Among the significantly enriched motifs identified based on genes showing significantly differential RNA expression and chromatin accessibility after HS, HSF-1, SNPC-4, CEBP-1, and HLH-30 stood out (Table 1, pink section). Specifically, the motif of HSF-1, a master regulator of the HS response, was enriched among the genes that showed upregulated RNA expression and chromatin accessibility after priming and HS, as well as those with downregulated chromatin accessibility post HS, perhaps reflecting the transient nature of some of the HSF-1-regulated gene expression [32]. SNPC-4, a component of the small nuclear RNA-activating complex, showed motif enrichment among genes upregulated after HS and regions with greater accessibility after priming. The motif of CEBP-1, a conserved bZIP transcription factor, was enriched among genes upregulated after priming. In contrast, the motif of HLH-30, the *C. elegans* ortholog of TFEB, was enriched among those downregulated after priming. RNAi knockdown confirmed that *hsf-1* is essential for heat hormesis in *glp-1(ts)*, as it completely abolished the enhanced survival to HS of the primed worms (Fig 4A). Intriguingly, knocking down *snpc-4* also significantly compromised the survival advantage induced by priming in *glp-1(ts)* (Fig 4B). However, no effects were detected with knockdowns of *cebp-1* or *hlh-30* under our hormesis regimen (S5 Data).

The motifs revealed by differential RNA-seq data included GATA transcription factors, ELT-2 and ELT-6, and a zinc finger transcription factor, PQM-1 (Table 1, green section). All three motifs were enriched among genes that showed downregulation after both priming and HS in the primed compared to naive groups. Interestingly, RNAi knockdown of *elt-2* completely abolished the HS survival advantage in primed *glp-1(ts)* worms (Fig 4C), while knockdowns of *elt-6* and *pqm-1* had no effect (S5 Data).

Several motifs were revealed based on differential ATAC-seq data (Table 1, blue section). Specifically, DPY-27, a homolog of the condensin-like protein and subunit of the dosage compensation complex (DCC), had its motif enriched in peaks downregulated after both priming and HS in the primed compared to the naive groups. Additionally, FOS-1, a c-Fos ortholog and AP-1 component, and ATF-7, a bZIP transcription factor, both had motifs enriched among peaks upregulated after HS. We observed that RNAi knockdown of *fos-1* consistently compromised the heat survival advantage of primed *glp-1(ts)*, but not the other candidate factors (Fig 4D and S5 Data). Together, these findings identify HSF-1, SNPC-4, ELT-2, and FOS-1 as putative mediators of the thermotolerance phenotype induced by our heat hormesis regimen in *glp-1(ts)*.

We next tested whether the new regulators of heat hormesis uncovered using *glp-1(ts)* also play similar roles in WT. Interestingly, RNAi knockdown of *hsf-1, snpc-4*, *fos-1,* and *mars-1* compromised the thermotolerance benefits of primed worms in the WT background (S7A and S7C–S7E Fig), suggesting that these regulators are broadly important for mediating the beneficial effects of heat hormesis. We note that *hsf-1* is critical for survival post HS in both naive and primed WT worms, as *hsf-1* RNAi knockdown drastically shortened their survival post HS. However, *hsf-1* RNAi knockdown did not completely eliminate the priming-induced survival benefits in WT (S7A Fig), unlike in *glp-1(ts)* (Fig 4A). Similarly, RNAi knockdown of *elt-2* did not impair priming-induced thermotolerance in WT (S7B Fig), unlike in *glp-1(ts)* (Fig 4C). In contrast, although *dpy-27* RNAi showed variable effects in the *glp-1(ts)* mutant, its knockdown consistently attenuated priming-induced thermotolerance in WT (S7F Fig). Together, these results highlight several putative regulators of heat hormesis with major effects in both WT and *glp-1(ts)*, while also point to factors with roles in specific genetic backgrounds.

### Transient germline defects and preserved HSP inducibility may underlie life span extension induced by heat hormesis

**Fig 5:**
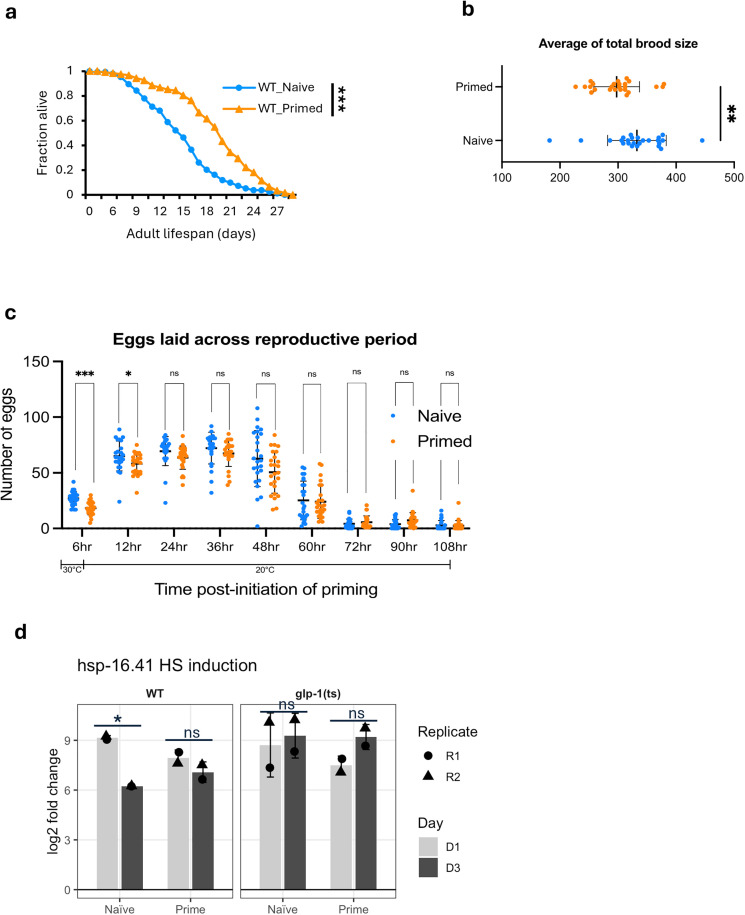
Heat priming induced life span extension, transient germline defects, and preserved HSP inducibility in WT. **(a)** Lifespans of WT worms with or without priming were assessed at 20 °C. Survival curves for WT_Naive and WT_Primed are shown. Data represent combined results from two independent experiments (*N* = 2). WT worms were maintained at 20 °C throughout their life cycle. **(b)** Average of total brood size, calculated from egg counts at nine time points across the reproductive period of individual worms, are shown in naive and primed groups. **(c)** Brood size was counted starting at the onset of priming (30 °C for the primed group, 20 °C for the naive group), with subsequent counts every 12 hours at 20 °C. **(b, c)** were performed in WT with *N* = 3 biological replicates and a total of 25 worms. **(d)** RNA induction of hsp genes (*hsp-16.41*) was quantified as log2 fold change (HS/before HS) using normalized RNA-seq counts from two biological replicates (R1, R2). Log-rank test was used to compare mean life span in **(a)**. Two-tailed unequal variances t-tests were performed in **(b, c)**, and paired t-tests were used in **(d)**. * *p* < 0.05; ** *p* < 0.01; *** *p* < 0.001. Details are provided in S1 Data.

Heat hormesis has been widely recognized for its longevity benefits, and previous studies have reported that WT worms exposed to the priming regimen used here (30 °C for 6 hours on day 1 of adulthood) exhibited a longer life span [6,16]. We confirmed this effect in WT; however, interestingly, the same regimen did not further extend life span in the long-lived *glp-1 (ts)* mutant (Figs 5A and S8A).

To investigate this difference, we first examined reproductive function, the major distinction between the two genetic backgrounds. Priming resulted in a small but significant reduction in total brood size in WT (Fig 5B), which was due to a significant decrease in laid embryos during the priming period (and the embryos laid during that period were also inviable), and a residual defect within 12 hours after priming, with egg laying and embryo viability returned to normal levels thereafter (Figs 5C and S8B).

To further understand how heat priming might impact WT worms, we re-examined the WormExp comparison results [33]. As described above, for the priming-induced upregulated genes, germline stem cell removal-associated genes and *glp-1(ts)-*upregulated genes ranked among the most significantly enriched categories (S1 Data). This finding corroborates the brood size results and indicates that germline proliferation is transiently suspended during heat priming.

Previous research showed that WT worms exhibit an attenuated heat stress response during early adulthood, evidenced by substantially lower induction of hsp genes, including *hsp-16* and *hsp-70*, in day 2 compared to day 1 adults in response to HS [34]. This is proposed to reflect maximizing germline activity rather than somatic stress responses during reproduction. To test whether heat priming affects this regulatory step, we examined the inducibility of hsp genes during early adulthood in primed and naive WT and *glp-1(ts)* worms using RNA-seq data of day 1 or day 3 adult worms after HS. Our data confirmed reduced *hsp* induction in WT at day 3 relative to day 1, consistent with previously published data [34], while *glp-1(ts)* did not show this early-adulthood decline (Fig 5D). Strikingly, we found that priming reversed this decline in *hsp* gene induction in WT, with primed day 3 adults maintaining *hsp* induction at levels comparable to day 1 adults (Fig 5D). These results suggest that heat priming preserves the inducibility of the HS response during the reproductive period in WT.

## Discussion

Our study provides a comprehensive view of the transcriptomic and chromatin accessibility dynamics across a heat hormesis regimen in both WT and germline-less *glp-1(ts)* mutant animals, revealing molecular evidence of dose-dependent effects in which varying levels of heat stress elicit distinct molecular responses. Although priming-induced RNA expression and chromatin accessibility changes largely restore after a recovery period, the earlier stress exposure leaves distinct molecular imprints with lasting physiological consequences. Notably, our comparative analysis of WT and *glp-1(ts)* animals demonstrates that germline responses during heat priming profoundly impact long-term physiology, resulting in significant extension of mean and median life span. Importantly, we identify several highly conserved regulators of heat hormesis, some acting broadly across both genetic backgrounds and others functioning in a context-dependent manner. These findings provide a molecular framework linking transient stress exposure and long-term physiological benefits, with important implications for understanding how mid-life stress management can promote healthy aging.

### Molecular evidence of dose-dependent effects

Our combined RNA-seq and ATAC-seq strategies proved complementary in uncovering molecular changes across heat hormesis. At both the level of individual genes showing changes in RNA expression and chromatin accessibility and at the level of biological functions enriched among various gene sets, our data provide compelling evidence that different levels of heat stress induce differential molecular responses. This aligns with the foundational concept of hormesis, in which a stressor generates dose-dependent effects [3,35]. Since we conducted a time-series analysis, it is possible that the dose-response changes we observed are influenced by the developmental stage of the worms subjected to priming versus HS. However, comparisons with published studies in which L4 or day 1 adult worms are exposed to 35 °C HS [29,30] reveal substantially greater overlap with our HS response (in day 2 worms) than with the priming response (in day 1 worms) (S3D and S5G Figs). This indicates that developmental timing plays a relatively minor role in the stress-induced molecular responses in our study.

### Modest correlation between RNA-seq and ATAC-seq

We observed a modest correlation between the RNA-seq and ATAC-seq datasets (S2C Fig), which is consistent with previous findings that ATAC-seq and RNA-seq results often do not correlate [36], reflecting different modes of gene regulation that may not be correlative. First, changes in chromatin accessibility and gene expression may occur at different times following heat stress, leading to temporal discordance between the two datasets. In particular, RNA-seq measures steady-state mRNA levels, which do not necessarily reflect active transcription at the time of sampling. In contrast, chromatin accessibility changes may more closely correlate with transcriptional activity. Second, not all gene expression changes are directly driven by alterations in chromatin accessibility. For example, transcription factors binding and local changes in chromatin could have consequential effects on gene transcription, without detectable changes in chromatin accessibility as accessed by ATAC-seq. Third, additional layers of regulation, such as post-transcriptional RNA modifications, RNA decay, or stress granule-mediated mechanisms, may also contribute. Finally, bulk sequencing approaches likely obscure tissue- or cell-type–specific responses, further contributing to the limited overall correlation between RNA-seq and ATAC-seq profiles. Future follow-up analyses, guided by limitations suggested above, including single-cell transcriptomics and genomic strategies that can distinguish immediate transcriptional responses to post-transcriptional regulation, will likely provide a more in-depth understanding of the gene regulatory landscapes across heat hormesis.

### New regulators of heat hormesis

**Fig 6:**
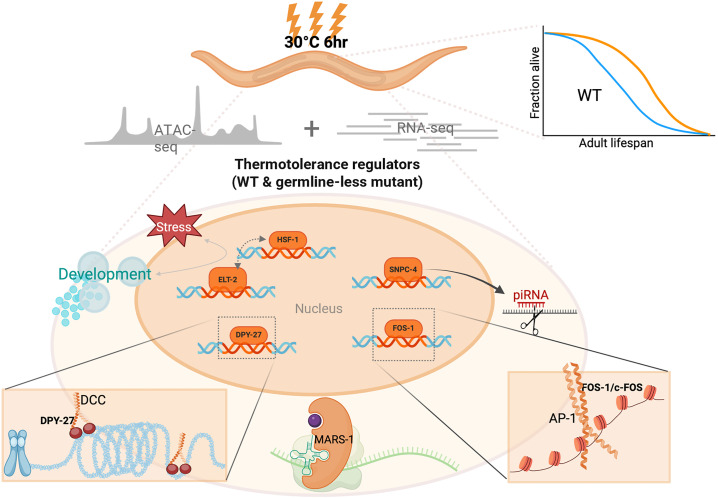
Heat hormesis engages specific regulators to induce thermotolerance in WT and *glp-1(ts)* mutant. Our study reveals that heat hormesis promotes longevity in WT and induces thermotolerance in both WT and *glp-1(ts)* mutant animals. Our multiomic data lead to the identification of several key regulators of heat hormesis, all of which are evolutionarily highly conserved, and participate in different regulatory steps of gene expression. HSF-1 is the master transcription factor of heat shock response and its emergence from our analyses proves our investigative strategy is effective. FOS-1 points to a potential role for the AP-1 pioneer transcription factor complex in encoding heat hormesis memory through chromatin remodeling. ELT-2 suggests an interaction between the germline and the intestine in stress adaptation. DPY-27 suggests a connection between the dosage compensation complex (DCC)-mediated chromosome architecture and heat stress management. HSF-1, ELT-2, and DPY-27 regulate heat hormesis differently in worms with or without germline. SNPC-4 implicates a role of piRNA-mediated post-transcriptional regulation in stress responses. MARS-1 implicates a role of methionine incorporation during protein synthesis in heat hormesis. Created in BioRender. Lee, S. (2026) https://BioRender.com/msaxe8u.

Our multiomic data, coupled with functional screens, provided critical entry points for identifying candidate regulators of heat priming-induced thermotolerance (Fig 6). **HSF-1** is the master transcription factor that is essential for mounting a HS response [37,38], and its emergence from our analyses is a strong proof-of-principle that our investigative strategy is effective in uncovering bona fide regulators of the heat stress response. An intriguing new finding from our data is that HSF-1 appears to act somewhat differently in mediating heat priming-induced thermotolerance in WT versus germline-less *glp-1(ts)* strains, where it is completely required in *glp-1(ts)* but only partially required in WT. Going forward, it will be particularly important to understand the spatial regulation of HSF-1, which may illuminate how its role in mounting a HS response in different cells can impact overall physiological outcomes of heat hormesis. Similarly, **ELT-2**, a GATA transcription factor, is critical for intestinal development and immunity in *C. elegans* [39–41]. Prior work demonstrated that ELT-2 is required for enhanced thermotolerance in HSF-1-deficient worms [42], suggesting the possibility that ELT-2 and HSF-1 act in complementary pathways. We found that ELT-2 is essential for heat priming-induced thermotolerance in *glp-1(ts)* mutants but dispensable in WT, highlighting a possible cross-tissue interaction between the germline and the intestine and positioning ELT-2 as a context-dependent regulator of hormesis. In contrast, we found that **DPY-27**, a homolog of human SMC4, a condensin-like subunit of the DCC, plays a consistent role in mediating thermotolerance of primed WT but not *glp-1(ts)*. In *C. elegans*, the DCC contributes to the formation of topologically associated domain (TAD) boundaries that regulate chromosome-wide gene expression, and elimination of DCC-dependent TADs on the X chromosome has been shown to reduce heat tolerance and shorten life span [43,44].

**FOS-1**, the *C. elegans* ortholog of c-Fos, a subunit of the activator protein-1 (AP-1) complex, is critical in mediating heat priming-induced thermotolerance in both WT and *glp-1(ts)*. Mammalian AP-1 is well-characterized to regulate diverse processes, including stress responses [45], and *C. elegans* FOS-1 is known to regulate hyperosmotic stress [46,47]. Importantly, with aging, mammalian AP-1 progressively shifts its occupancy from chromatin sites linked to developmental genes to those involved in stimuli and stress responses, resulting in remodeling of chromatin accessibility due to its pioneer transcription factor activity [48,49]. The age-dependent AP-1 redistribution on the chromatin was speculated to reflect a type of “epigenetic memory,” which may align with our findings, where FOS-1 DNA motif is specifically enriched in more open chromatin regions in primed worms, suggesting its role in maintaining stress memory through chromatin opening in heat hormesis. This finding corroborates recent studies highlighting the roles of the SWI/SNF chromatin remodeling complex and the histone acetyltransferase CBP-1 in maintaining persistent induction of innate immune genes, thereby mediating stress adaptation and longevity following early-life heat exposure [21].

**SNPC-4,** a subunit of the small nuclear RNA-activating complex (SNAPc), which is essential for piRNA transcription in *C. elegans* [50,51]. Interestingly, previous studies suggest that high temperatures suppress piRNA biogenesis [52]. Moreover, consistent with a possible role of piRNAs in heat hormesis, *prg-1*, which encodes a Piwi-family Argonaute protein that binds piRNAs [53], is among one of the few genes that showed persistent upregulation through heat hormesis (S1 Data). These findings implicate post-transcriptional regulation in heat hormesis. **MARS-1** is a conserved methionine tRNA synthetase. *mars-1* knockdown enhances thermotolerance of naive worms, and heat priming only confers marginal further protection. Interestingly, human MARS1 mutations have been implicated in chronic activation of the integrated stress response [54], suggesting conserved roles in stress response from worms to humans. The identification of MARS-1 from our study implicates a possible link between methionine incorporation during protein synthesis and priming-induced protective effects.

Together, the putative new regulators of heat hormesis identified here point to different steps of gene expression regulation in mediating the beneficial effects of heat hormesis. These regulators are highly conserved, and their further characterization promises to unveil mechanistic principles that can be broadly relevant to stress adaptation and healthspan regulation. Another intriguing consideration is how these new regulators enable long-term physiological benefits. Future investigation to probe whether these factors are regulated post-transcriptionally, translationally, and/or post-translationally will provide informative insights about how “memory” of the earlier heat priming can persist.

### Germline-dependent mechanisms of heat hormesis

Our complementary studies in WT and *glp-1(ts)* led to the intriguing finding that heat hormesis likely extends life span through a germline-dependent mechanism. Multiple lines of evidence support this conclusion: First, heat priming only extends life span in WT, not in germline-less *glp-1(ts)* mutants. Second, heat priming transiently disrupts reproduction. Third, heat priming induces gene expression changes in WT animals that strongly resemble those associated with germline ablation. Fourth, heat priming preserves hsp gene inducibility upon HS during early adulthood, similar to the effect of *glp-1(ts)* mutation. Notably, while germline stem cell ablation or its genetic mimic, such as the *glp-1(ts)* mutation, is well-established to extend life span in *C. elegans* [55] and other diverse species [56–58], our findings demonstrate that temporary, transient disruption of germline activities in response to heat stress produces a prolonged and significant effect on physiological effects. Furthermore, our data reveal that specific regulators of heat hormesis, such as HSF-1, ELT-2, and DPY-27, regulate stress adaptation in a genetic background-dependent manner. These results have broader implications: whereas hormetic stress can enhance stress resilience and longevity, its benefits may be intertwined with trade-offs in reproductive or developmental functions. Future research to further investigate these complex relationships at the molecular level, especially with spatial resolution, will be critical for developing stress management strategies that promote healthy aging without compromising quality of life in the short term.

We additionally observed that our heat hormesis regimen primarily extends mean and median life span rather than maximum life span and induces a slight “squaring” of the survival curve (Figs 5A and S8A), indicating a significant reduction of early- and mid-life mortality. Importantly, this pattern resembles findings from worms experiencing a higher oxidant state during development [59], suggesting that lowering early- and mid-life mortality and improving mean and median life span, likely reflecting improved healthspan, may represent a universal signature of stress hormesis, independent of the specific stressor. Elucidating how different stressors confer common as well as unique stress responses and adaptations will be a critical area of future research that will inform potential health-promoting interventions via stress exposure and management.

## Methods

### C *elegans* maintenance

Unless otherwise specified, worms were maintained on 6-cm NGM plates seeded with 0.2 ml of a five-times concentrated overnight culture of streptomycin-resistant OP50 bacteria (live OP50). 10-cm NGM plates containing 1 ml of a 25-times concentrated overnight culture of streptomycin-resistant OP50 were used for maintaining larger amounts of worms. Killed OP50 bacteria were prepared by resuspending live OP50 in LB containing 100 µg/ml Carbenicillin and 15 µg/ml Tetracycline twice, then concentrating it 20-fold. The bacteria incubated with the antibiotic on a rocker for at least 30 min before use. Killed OP50 was used to mitigate bacterial infections in *glp-1 (ts)* mutant strains (*e2144* temperature-sensitive allele) during survival assays. The N2 strain was maintained at 20 °C, while the *glp-1 (ts)* mutant strain was maintained at 16 °C unless noted differently.

### Growth of synchronized worms on a small scale for phenotypic experiments

For the **WT** N2 strain: Several plates were set up with 4−5 gravid adults per plate for egg laying at 20 °C for 3−4 hours. After egg laying, gravid adults were removed and eggs were incubated at 20 °C for approximately 65 hours. The synchronized gravid adults were then distributed to fresh plates, approximately 30 per plate, before the experiments began. Priming was performed at this time when the N2 worms had just reached the gravid adults of their Day 1 (D1) adult stage. **For the *glp-1(ts)* mutant strain:** Several plates were set up with 6−8 gravid adults per plate for egg laying at 16 °C for 4−5 hours. The gravid adults were removed after egg laying, and the eggs were incubated at 25 °C for approximately 48 hours until they became young adults and were transferred to 20 °C (“Young adult” refers to the developmental stage immediately after the L4 stage, in which the worm has molted into a reproductive adult but has not yet begun laying a large number of eggs). After incubating overnight at 20 °C (approximately 15−17 hours), the synchronized adults were distributed to fresh plates with killed OP50, approximately 30 per plate, before starting experiments. Priming was performed at this time when sterile *glp-1(ts)* worms were approximately at their D2 adult stage. The WT N2 was sometimes conducted in parallel as a positive control, following the same maintenance protocol as *glp-1(ts).*

### Priming and HS experimental setup

Worms were subjected to incubators with setting pre-adjusted to target temperatures (30 °C for 6 hours for priming, and specific temperatures and duration for HS depending on the experiment). During incubation, the lids of the plates were replaced with those having manually drilled holes, and the plates were wrapped with parafilm on the sides. The plates were placed on a metal rack in the incubator with agar side facing down. This setup aimed to allow worms to reach the target temperature more efficiently and to maintain consistent temperature and humidity inside the plates. After temperature treatments, the parafilm was removed, and the lids were switched back to the normal ones without holes before being moved back to 20 °C.

### Thermotolerance assay

To explore how priming affected thermotolerance, defined as regaining motility after exposure to the indicated HS temperature and duration, we conducted tests. For small-scale phenotypic experiments, 30–40 worms were placed on each 6-cm plate, with at least two plates per group for each biological replicate. For large-scale experiments, approximately 1,000 worms were placed on each 10-cm plate. After being subjected to HS at 35 °C for 10 hours or other specified temperatures and durations (detailed in S1 Data), worms were maintained at 20 °C until scoring. Scoring occurred 15 hours post-HS, with each plate being tapped 10 times consistently immediately before scoring. For small-scale experiments, worms that moved without being touched by a picker were counted, along with the total number of worms, to calculate the percentage of moving worms. For large-scale experiments, a consistently marked 1/9 area of each 10 cm plate was used to count worms that moved without being touched and the total worms within the marked area to calculate the percentage of moving worms. Three separate areas were marked and scored for each experimental group as technical replicates. Three independent experiments were conducted as biological replicates. Two-tailed unequal variances *t* test was performed to determine if the differences between primed and naive groups were significant.

### Survival assay after HS

Synchronized worms were subjected to priming, recovery, and HS or were maintained without priming for the naive group, according to specific temperatures and durations as detailed in S1 Data. After HS, worms were scored every two days until all had died. Worms were scored as dead when they failed to respond to a gentle prod on the head by a worm picker. Worms were maintained at 20 °C after HS and throughout the experiments. Data were analyzed using OASIS2 online survival analysis tool [60]. The Kaplan-Meier estimator was used to re-plot survival curves in Excel, with the ‘Fraction alive’ on the y-axis and days after HS on the x-axis. Log-rank tests were utilized to determine if there were significant differences in survival times between the two groups. All survival assay experiments were conducted independently at least twice. See S1 Data for raw data and analysis details.

### Lifespan

Synchronized worms were subjected to priming, or were maintained without priming for the naive group, at the indicated timing mentioned above and detailed in S1 Data. Worms were scored subsequently every two days until all had died. Worms were scored as dead when they failed to respond to a gentle prod on the head by a worm picker. For the WT N2 strain, worms were transferred to fresh plates every 1−2 days until the end of their reproductive period. For the *glp-1(ts)* mutant strain, worms of both primed and naive groups were transferred to plates with killed OP50 before the priming experiments. Data were analyzed using OASIS2 online survival analysis tool as mentioned above. The Kaplan–Meier estimator was used to re-plot survival curves in Excel, and log-rank tests were utilized to determine if there were significant differences in survival times between the two groups. All life span assay experiments were conducted independently at least twice (see S1 Data for raw data and analysis details).

### Reproductive function experiments

WT N2 worms were maintained and age-synchronized at 20 °C. After approximately 65 hours of development from eggs, newly matured gravid adults were distributed, one per 3.5 cm plate. The primed group was subjected to a 6-hour priming at 30 °C and returned to 20 °C, while naive group was kept at 20 °C in parallel during priming period. After priming, each worm was transferred to a new 3.5 cm plate to continue laying eggs, and the eggs left on the original plate were counted. The worms were subsequently transferred to new plates every 12 hours until they stopped laying eggs (~5 days). Once counted, the eggs were incubated at 20 °C to allow for hatching. Larvae were then counted 24 hours later. In total, 9 data points for both egg and larvae counts were collected. The average total brood size was calculated by summing egg counts from 9-timepoints across the reproductive period of 25 individual single worms. The average brood size for each time frame (6 hours for the first time point and 12 hours for the subsequent eight timepoints) was calculated across these 25 worms. The average hatching rate for each timepoints was calculated by dividing the number of eggs by the number of larvae for 25 worms. Hatching rates were adjusted to 1 if the values exceeded 1 to account for potential miscounts of eggs, which might be less visible than larvae during counting. Data of 25 worms were collected across three independent experiments, with each biological replicate containing around 5 ~ 10 worms per group. Details can be found in S1 Data.

### RNA interference

HT115 bacteria expressing double-stranded RNA against the gene of interest were obtained from the Ahringer [61] or Vidal [62] libraries and verified by Sanger sequencing. RNAi bacteria and the control empty vector (E.V.), L4440, were cultured overnight in LB containing 100 µg/ml carbenicillin and shaken at 37 °C for 3–4 hours until an optical density of 0.6–0.8 was reached. The cultures were then induced with 1mM of IPTG and continued to incubate on a shaker at 37 °C for an additional 2.5 hours. After induction, the bacteria were centrifuged at 2,000 rpm for 15 min at room temperature and concentrated 50-fold. 200 µl of concentrated bacteria were seeded on 6-cm ‘RNAi plates”, which are NGM plates modified by omitting streptomycin and adding 100 µg/ml ampicillin, 15 µg/ml tetracycline, and 1mM IPTG. RNAi was administered to worms through feeding. The timing of RNAi administration varied depending on the genes, considering any developmental phenotypes caused by knocking down the gene. Details of the RNAi administration timing for each gene are documented in S5 and S6 Data. To determine whether RNAi knockdown of a specific gene compromised the priming-induced phenotype in survival extension, the survival extension rate was calculated using the formula:


(Mean survival days in primed − Mean survival days in naive)/Mean survival days in naive * 100

This measure quantifies the change in mean survival days in the primed group relative to naive in each independent experiment. A paired two-tailed *t* test was employed to determine statistical differences in survival extension rates.

### Collection of timed samples synchronized worms on a large scale for sequencing

Mixed-stage worms were collected into centrifuge tubes from several 6-cm NGM plates using M9 buffer with 0.05% TWEEN20. After allowing the adults to settle to the bottom of the tube for 30–60 s, the supernatant containing larvae was removed. This strategy was used to separate adults and larvae by washing worms several times until the tube contained mostly adults. Adults in 1 ml M9 buffer with 0.05% TWEEN20 were quantified and distributed to new plates for egg laying.

1,000–2,000 adults were placed on 10-cm plates and incubated at 16 °C for 4–5 hours (for *glp-1 (ts)*) or 20 °C for 3–4 hours (for WT) for egg laying. 5 ml M9 buffer was gently applied to wash off adult worms from the plates without disturbing the bacteria lawn, repeating until most of the adults were removed. 5 ml M9 with 0.05%TWEEN20 was applied to wash off the bacterial lawn containing embryos using the liquid force generated by repeated pipetting. The liquid containing embryos was collected into centrifuge tubes and spun down for one minute at 2,000 rpm. After removing the supernatant, the pelleted embryos were resuspended thoroughly in M9 with 0.05% TWEEN20. The synchronized embryos were quantified and distributed at around 1,200 embryos per 10-cm plate. At the assigned timepoints, synchronized worms were washed off and collected into centrifuge tubes using M9 with 0.05% TWEEN20. After the worms settled in the bottom of the tubes, the supernatant was removed and worms were transferred to 1.7 ml low-binding tubes. Worms were washed in M9 buffer 1–5 times until the supernatant was clear, indicating most of the bacteria were removed. For RNA-seq, 200 worms were aliquoted to a low-binding tube and snap-frozen in 1 ml TRIZOL using liquid nitrogen after removing most of the M9 buffer. The remaining 1,000 worms were snap-frozen, after removing most of the M9 buffer, in liquid nitrogen for ATAC-seq. Snap-frozen samples were stored at −80 °C until further processing.

Five biological replicates for RNA-seq (labeled R_r1, R_r2, R_r3, R_r4, R_r5) and four for ATAC-seq (labeled A_r1, A_r2, A_r3, A_r4) were processed in two independent sequencing batches. For RNA-seq, R_r1, R_r2, and R_r3 were in the first batch, and R_r4 and R_r5 in the second. For ATAC-seq, A_r1 and A_r2 were in the first batch, and A_r3 and A_r4 in the second. Each replicate includes samples from multiple timepoints across our regimen in both primed and naive groups (Fig 1A). Data from all timepoints have at least three biological replicates, except for T3.6, which has two biological replicates in ATAC-seq.

### RNA extraction and RNA-seq

200 snap-frozen worms per sample were homogenized by alternating between thawing, vortexing, and refreezing in liquid nitrogen four times. 200 µl chloroform was added to each tube and samples were vortexed for 15 seconds, followed by incubation at room temperature for 3 min and centrifugation at 12,000 *g* for 15 min at 4 °C. The upper aqueous layer was transferred to a fresh tube, and 500 µl of isopropanol was added to each sample. After adding 1 µl of GlycoBlue and incubating for 15 min at room temperature, samples were centrifuged at 12,000 *g* for 10 min at 4 °C. The supernatant was discarded, and the RNA pellet was washed with 1 ml of 80% ethanol. All remaining ethanol was removed after centrifugation at 12,000 *g* for 10 min at 4 °C, and the RNA pellet was air-dried for 5–7 min and then dissolved in 17 µl of DEPC water. The RNA was then treated with DNase to remove residual DNA, following the instructions from the TURBO DNase kit (Invitrogen AM1907). After DNase treatment, the RNA was further purified using an RNA cleanup kit (ZYMO Research R1015). RNA concentration was measured using the Qubit RNA HS Assay kit (ThermoFisher Q32851). Five biological replicates were processed as described above.

RNA-seq libraries were prepared using the QuantSeq 3′ mRNA-Seq Library Prep Kit (Lexogen FWD 015). For replicates R_r1, R_r2, and R_r3, the preparation started with 100 ng of RNA from each sample and employing 15 PCR cycles. For replicates R_r4 and R_r5, the libraries were prepared starting with 70 ng of RNA and employing 17 PCR cycles. The libraries were quantified using a Qubit DNA HS Assay kit and their quality was assessed with a Bioanalyzer. Subsequently, the libraries were submitted for single-end 86 bp sequencing on an Illumina NextSeq 500 machine. Replicates R_r1, R_r2, and R_r3 were pooled in one sequencing lane, while R_r4 and R_r5 were pooled in another, with each pool sequenced in separate runs.

### RNA-seq data analysis

#### Upstream analysis.

Adaptor sequences were trimmed, and low-quality reads filtered from raw fastq files using the trim_galore function in Cutadapt (version 4.6) with settings -q 20 --fastqc. Trimmed sequencing reads from the FASTQ files were then aligned to the reference genome (ce11/WBcel235) using STAR aligner. Initially, the reference genome was indexed with the following settings: --runThreadN 8 --runMode genomeGenerate --genomeDir [genome_directory] --genomeFastaFiles [genome fasta_file] --sjdbGTFfile [gtf_file] --sjdbOverhang [read length – 1]. Alignment was performed using STAR settings: --quantMode GeneCounts --genomeDir [genome_directory] --readFilesIn [trimmed sequencing file] --readFilesCommand zcat --runThreadN 2 --outFileNamePrefix [output_file_prefix] --outFilterMultimapNmax 1 --outFilterMismatchNmax 2 --outSAMtype BAM SortedByCoordinate. The resulting tab-delimited text files, named with the prefix “_ReadsPerGene.out.tab”, contained counts for reads aligned to the plus strand of RNA in column 3, which is recommended and applied for 3′ RNA-seq data. Counts from each sample were aggregated to generate a matrix that was uploaded to RStudio for downstream differential gene expression analysis (S9 Data).

Total aligned reads and the percentage of uniquely mapped reads from all sequencing files were accessed. Only uniquely mapped reads were utilized for downstream analysis (S1 Data: RNA-seq QC). The correlation of aligned reads across biological replicates was calculated using Spearman correlation and visualized with a heatmap using Morpheus (https://software.broadinstitute.org/morpheus). (S1A–S1E Fig). The color scheme of the heatmap displays a minimum value of 0.85 and a maximum value of 1.

#### Differential RNA expression analysis.

A matrix (S13 Data) containing raw read counts was generated and uploaded to RStudio for DESeq2 analysis. The matrix was subsetted according to timepoints and replicates for further comparison. For comparisons involving timepoints 1, 2, and 3.3, columns including five replicates (R_r1, R_r2, R_r3, R_r4, and R_r5) for samples (NC, N1, P1, N2, P2, N3.3, and P3.3) were subsetted. For comparisons involving time point 3.6, columns including three replicates (R_r1, R_r2, and R_r3) for samples (NC, N1, P1, N2, P2, N3.3, P3.3, N3.6, and P3.6) were used. Prior to differential analysis, read counts were normalized using the estimateSizeFactors function. The normalized counts were then filtered to retain only genes with more than 5 normalized counts in at least the minimum number of replicates per sample. To better estimate log2 Fold Change (log2FC) for genes with low counts and high dispersion, the apeglm method was employed for shrinkage during differential analysis. Genes with adjusted p-values less than 0.05 were considered significant. MA and Volcano plots were generated using methods described in the ATAC-seq analysis section (S9 Data).

### Nuclei purification and ATAC-seq

Worms, previously snap-frozen and stored in low-binding tubes, were thawed on ice. All subsequent steps were conducted on ice or at 4 °C, with all materials pre-chilled to maintain nuclei integrity. An equal volume of freshly prepared Nuclei Purification Buffer (NPB) [63] (recipe available in S1 Data: ATAC protocol) was added to the worm pellet. The worms were homogenized manually five times using Pellet Pestles coated with Fetal Bovine Serum. The homogenization frequency was optimized to minimize damaging nuclei. After homogenization, worm pellets were allowed to settle for 3–5 min, then centrifuged at 100–200 *g* for 3 min. The supernatant, containing the nuclei, was transferred to a fresh low-binding tube. The remaining worm pellets were resuspended in an equal volume of 2× NPB and homogenized again as described. This process was repeated for multiple rounds until no visible worm pellet remained, typically requiring 7–8 rounds. The first six rounds involved homogenizing five times, with the final rounds reduced to three times to preserve nuclei integrity. After completing the homogenization, the collected supernatants were centrifuged at 100–200 *g* to remove debris, transferring the clean supernatant into a new tube. A subset of nuclei was stained with DAPI and examined under a fluorescence microscope to assess quality. Nuclei counts were determined using a hemocytometer with DAPI staining. For ATAC-seq, 50,000 nuclei per sample were aliquoted into a standard tube (non-low-binding) and pelleted by centrifugation at 1,000 *g* for 10 min. The pellet location was marked, and the supernatant was carefully removed to avoid contamination with bacterial DNA.

Purified nuclei were immediately subjected to the ATAC-seq procedure. Nuclei were gently resuspended in 47.5 µl Omni-ATAC buffer (composed of 2× Illumina Tagment DNA (TD) buffer, 16.5 µl 1× PBS, 0.1% TWEEN20, and 0.01% Digitonin) and mixed with 2.5 µl of Illumina Tagment DNA Enzyme (TDE1) (Cat#20034197). The mixture was incubated at 37 °C on a thermomixer set to 500 rpm for 0.5–1 hour. Tagmented DNA was then purified using a MinElute PCR Purification Kit (Qiagen #28004) and eluted in 10 µl. The purified DNA fragments were stored at −20 °C until PCR amplification.

PCR amplification was performed using NEBNext Ultra II Q5 Master Mix (M0544S) in a total volume of 50 µl, with 9 µl of purified tagmented DNA and 25 µM primer concentration. The thermocycling protocol included an initial step at 72 °C for 5 min, followed by 98 °C for 30 s, and 12 cycles of 98 °C for 10 s, 63 °C for 30 s, and 72 °C for 1 min. Each sample utilized a common Adapter 1 primer (Ad1) and a unique index Adapter 2 (Ad2*) primer.

Amplified ATAC-seq DNA libraries were purified using the MinElute PCR Purification Kit (Qiagen #28004) and eluted in 25 µl of warm DEPC water. Libraries were size-selected to retain DNA fragments between 100 and 600 bp using Blue Pippin service, quantified using a Qubit, and quality-checked using a Bioanalyzer. Sequencing was performed on an Illumina NextSeq 500 machine, with libraries from replicates A_r1 and A_r2 pooled at a final concentration of 3.6 ng/µl for one sequencing lane, and libraries from replicates A_r3 and A_r4 pooled at a concentration of 1.96 ng/µl for another lane. Each pool was sequenced independently. More details about nuclei purification and ATAC-seq can be found in S1 Data: ATAC protocol.

### ATAC-seq data analysis

#### Upstream analysis: From raw data to final BAM files:.

The paired-end FASTQ files, denoted as “.R1” and “.R2”, were organized for processing. Adapters were trimmed using Cutadapt (version 4.6) integrated with Trim Galore, applying the parameters --gzip -nextseq 20 --cores 8 --paired. The trimmed, paired-end sequencing files were then aligned to the reference genome *Caenorhabditis elegans* WBcel235 (ce11) using BWA with settings -t 8 -M. Following alignment, SAM files were converted to BAM format and subsequently sorted and indexed using SAMtools. To refine the BAM files further, mitochondrial DNA (mtDNA) sequences were excluded using samtools view. Sequences listed in the *Caenorhabditis elegans* ce11 blacklist (version 2) were also removed using bedtools intersect. Duplicate reads were marked using Picard Tools’ MarkDuplicates and excluded with samtools view -F 0X400. The resultant BAM files, purged of mtDNA, blacklist regions, and duplicates, were indexed for downstream analysis.

#### Quality control (QC) and Transcription Start Site (TSS) enrichment analysis.

QC metrics were assessed using several tools. Aligned and uniquely mapped reads were quantified using samtools view -c, while samtools idxstats provided percentages of mtDNA reads. Duplicate read percentages were determined using Picard Tools’ MarkDuplicates function and summarized in MultiQC reports (S1 Data: ATAC-seq QC). For TSS enrichment analysis, the DeepTools suite was utilized. BAM files were first converted to BigWig format with bamCoverage, which translates raw read counts into coverage tracks. This step was performed without scaling adjustments to preserve original read densities. TSS signal coverage was then calculated for regions spanning 1,000 bp upstream and downstream of each TSS using the computeMatrix command (settings: reference-point -a 1000 -b 1000). Heatmaps visualizing these coverage intensities were generated with plotHeatmap. All samples demonstrated enriched reads at TSS, aligning with a distinct peak at each TSS. The reference TSS data, adopted from Chen and colleagues [64], was based on transcription initiation cluster “mode positions” mapped to the ce10/WS220 genome. To ensure compatibility with our analyses, which utilized the ce11 reference genome, TSS data was converted using the UCSC LiftOver Tool.

#### Peak calling.

Following QC of the upstream analysis, final indexed BAM files were utilized for narrow peak calling. Each sample and replicate underwent peak calling independently using MACS2 (version 2.2.7.1-r9). The settings employed were -f BAMPE --bdg --SPMR --gsize ce -q 0.05 --call-summits, with the default local correction parameters set to --slocal 1000 --llocal 10000. For visualization of read coverages, the makeTagDirectory function from the Homer software suite was used to create tag.dir. Bedgraph files were then generated using the makeUCSfile function with the parameters.tag.dir -o auto -fsize 1e10 -res 1 -color 106,42,73 -style chipseq.

#### Identifying “consensus peaks” and quantification of reads in peaks.

To identify “consensus peaks” for differential chromatin accessibility analysis, a three-step process was implemented:

1. **Merging read files:** BAM files from all biological replicates within each experimental group were merged using the samtools merge command. Experimental groups included NC, N1, N2, N3.3, P1, P2, P3.3 with four biological replicates each, and N3.6, P3.6 with two biological replicates each.
2. **Calling consensus peaks:** Using MACS2, consensus narrow peaks were called from a single command line that included all merged BAM files as inputs, using settings previously described.
3. **Subdividing peaks based on summits:** The identified consensus narrow peaks were further subdivided based on their summits using the splitMACS2SubPeaks.perl script adapted from Daugherty and colleagues [63]. This subdivision produced a total of 30,404 ′Consensus Peaks’ with an average length of 481 bp. The genomic regions of the 30,404 Consensus Peaks, labeled with the PeakID syntax ‘4reps.HS6hr_ConsensusPeaks_peak_’, are available in S1 Data.

Quantification of reads in peaks: Reads within these “consensus peaks” for each individual sample were quantified using the featureCounts program, facilitating subsequent analyses of chromatin accessibility variations across samples.

#### QC and consistency of biological replicates.

FRiP scores, representing the fraction of mapped reads in identified region of consensus peaks, were calculated for each sample using the featureCounts program and aggregated with multiqc. Across all samples, FRiP scores ranged from 31% to 49%, indicating no significant variance among biological replicates and suggesting reliable peak calling and reproducibility across different batches (S1K Fig). To further assess the consistency of biological replicates, genome-wide correlations of mapped reads between biological replicates were analyzed using the bedtools multicov function with a 2 kb sliding window across the ce11genome, segmented by the bedtools makewindows function. Read counts from each BAM file in each 2 kb window were logged (log10 transformation) and uploaded into RStudio. Pearson’s correlation coefficients between each replicate were calculated using the cor function in R, compiled into a matrix, and visualized using the heatmap.2 function from the gplots package in R, categorizing samples by timepoints for clarity in correlation assessment (S1F–S1J Fig). Details can be found in S7 Data.

#### Differential analysis of chromatin accessibility.

A matrix containing raw read counts in consensus peaks was generated and uploaded to RStudio for DESeq2 analysis. The matrix encompassed four biological replicates (A-r1, A-r2, A-r3, A-r4) for samples: NC, N1, P1, N2, P2, N3.3, and P3.3 and two biological replicates (A-r1, A-r2) for the samples: N3.6 and P3.6. (S14 Data). This matrix was loaded into RStudio and served as input for DESeq2 for differential analysis. Prior to analysis, read counts were normalized using the estimateSizeFactors function. Only peaks with more than five normalized counts in at least two samples, the minimum number of replicates in some experimental groups, were retained for analysis. To refine the estimation of log2FC for peaks with low counts and high dispersion, the apeglm method was utilized for shrinkage during the differential analysis. Peaks with adjusted p-values below 0.05 were determined significant. MA and Volcano plots were generated via ggplot2, where peaks significantly upregulated are marked in red and those downregulated in blue, unless labeled specifically. The R script for this analysis can be found in S9 Data.

#### Peak annotation.

For annotating consensus peaks, a dataset of 42,245 accessible elements, termed Reference Elements, derived from the study by Jänes and colleagues [65] (elife-37344-fig1-data1-v2) was utilized. These Reference Elements, adapted using ce11 genome information (S15 Data), facilitated the annotation of consensus peaks, which involved matching consensus peaks to Reference Elements with at least a 50% overlap. This annotation process was conducted using the ‘findOverlapsOfPeaks’ function within the ‘ChIPpeakAnno’ R package (S10 Data). A total of 30,404 consensus peaks were annotated, linking them to 19,352 functional information records that included associated genes and functional elements such as promoters and enhancers (S1 Data: annotated.ConsensusPeaks).

### Motif analysis

Motif enrichment analysis was performed using the Simple Enrichment Analysis (SEA) tool from THE MEME Suite (version 5.5.7) [66]. To identify motifs enriched in the promoter regions of significant genes from RNA-seq data, promoter sequences were obtained from the regions either 500 bp or 2,000 bp upstream of the TSS (S11 Data). The input sequences comprised promoter sequences from significant genes, while control sequences were promoter sequences from non-significant genes. For motifs enriched in significant peak regions from ATAC-seq data, BED files composed of significant peak regions were used as input sequences, while non-significant peak regions were used as control sequences.

The “JASPARA (non-redundant)-nematode2022 [67]” and “CIS-BP 2.0 Single Species-Caenorhabditis_elegans [68]” motif databases were applied to identify motif enrichments. Enrichment was considered significant if the *Q*-value was less than 0.05. Significantly enriched motifs were selected as candidates for RNAi screening if their associated genes showed detectable expression levels in our RNA-seq data. We primarily selected motif candidates identified in the JASPARA database for our RNAi screening, as the database has undergone more functional validation compared to the motifs in CIS-BP. Similar motifs were detected in both databases, while the HSF-1 motif was found exclusively in CIS-BP and not in JASPAR. The motif list can be found in S1 Data.

### Temporal dynamic analysis and trajectory plotting

Temporal dynamic analysis was performed in R using the log2FC values between prime and naive groups across all timepoints (S12 Data). Clustering was performed using the K-means algorithm (dtwclust), grouping genes into eight clusters based on similar temporal dynamic patterns. Euclidean distance was used in clustering to highlight shared trends over time. Mean expression profiles for each cluster were calculated and visualized using ggplot2 to display cluster-specific temporal patterns. Additionally, individual cluster plots were generated using highcharter to provide a dynamic view of each gene’s temporal trajectory within each cluster. The code is openly available on our GitHub repository (https://github.com/Rathalodusk/TimeSeriesClus) and on Zenodo (https://doi.org/10.5281/zenodo.18239265).

### Statistical information

Statistical analyses for thermotolerance and reproductive function experiments were performed using Microsoft Excel or GraphPad. Lifespan and survival assays after HS were analyzed using OASIS 2 [60]. GO analysis utilized WormCat 2.0 [69]. Additional statistical analyses, including Fisher’s exact test, were conducted in RStudio. The software and R packages employed for processing sequencing data and performing differential analyses in ATAC-seq and RNA-seq datasets are detailed in the Methods section. All experiments were conducted with at least two biological replicates, with similar results. Details regarding the number of replicates, sample size, types of statistical analyses, p-value cutoffs, and raw data are provided in the corresponding figure legends and/or supplementary data files.

## Supporting information

S1 Text Supporting details regarding RNA-seq/ATAC-seq quality and temporal trajectories of RNA expression change between primed and naive worms. Referred to as S1 Text in the main text.
(DOCX)

S1 Fig Quality control for transcriptomic and chromatin accessibility profiles in glp-1(ts). Spearman’s correlation analysis of RNA-seq profiles for both naive and primed groups of *glp-1(ts)* at timepoints 1 (**a**), 2 (**b**), 3.3 (**c**), 3.6 (**e**) and negative control (**d**) across independent replicates. Pearson’s correlation analysis of ATAC-seq profiles for both naive and primed groups at timepoints 1 (**f**), 2 (**g**), 3.3 (**h**), 3.6 (**i**), and negative control (**j**) across independent replicates. (**k)** Fraction of Reads in Peaks (FRiP) scores for individual samples, calculated using MultiQC based on featureCounts. “Assigned featureCounts” indicates mapped reads counted within identified consensus peaks (S1 Data). “Unassigned: no Features” indicates mapped reads not counted in consensus peaks. The FRiP scores within the identified consensus peaks ranged from 31% to 49% across all samples, affirming the good quality of the data. Furthermore, the FRiP scores among biological replicates were highly consistent, further supporting the reproducibility of our datasets. (**l)** TSS enrichment for all experimental groups in a representative biological replicate (r1): The top panel displays profile plots aggregating read coverage within 1kb upstream and downstream around TSS for all genes across the genome. The bottom panel displays heatmaps showing individual gene coverage, with each row corresponding to the TSS of a single gene, extending 1kb upstream and downstream. Colors in the heatmap indicating the level of read coverage. The plots for the remaining replicates can be found in S1 Data. Referred to as S1 Fig in the main text.
(TIF)

S2 Fig Differential analyses across timepoints and correlation between RNA expression and chromatin accessibility in *glp-1(ts).* MA plots display log2FC of gene expression from RNA-seq (top panel) and chromatin accessibility from ATAC-seq (bottom panel) for the indicated comparison in naive **(a)** and primed **(b)** groups. Differential analyses were calculated using DESeq2. Significant changes (*p*-adj < 0.05) are marked in red for upregulation (log2FC > 0) and in blue for downregulation (log2FC < 0), while unchanged are marked in gray (*p*-adj >= 0.05). Numbers indicate the count of significant genes for RNA-seq data and significant peaks (and their associated genes in brackets) for ATAC-seq data for each plot. **(c)** Scatter plots display genes with significant changes identified in either RNA expression or chromatin accessibility for the indicated comparisons. Genes with correlated changes between RNA-seq and ATAC-seq data are highlighted in purple based on defined filter criteria: log2FC RNA expression > 0.5, <−0.5; log2FC Chromatin accessibility > 0.25, <−0.25. These genes showed upregulation in both RNA expression and Chromatin accessibility or downregulation in both RNA expression and Chromatin accessibility. Genes without correlated changes are in gray. (**d)** Venn diagrams display the number of significantly differentially expressed genes (top panel) or peaks (bottom panel) for the 3-hour HS (light gray) and their overlap with the 6-hour HS (dark gray) in the indicated comparison. Substantial overlaps suggest that 35 °C heat shock for 3 or 6 hours elicited many similar changes in RNA expression and chromatin accessibility. **(e)** Venn diagrams display the number of heat shock/stress (HS)-induced genes identified from RNA-seq, comparing our data (N3.3 vs. N2, S3A Fig) with two published datasets (Schreiner and colleagues and Xu and colleagues). Details of experimental setup and HS conditions can be found in S1 Data. Fisher exact tests were conducted to determine if the overlap between this study (This paper) and published datasets are statistically significant. *** Indicates *p* < 2.2e−16. Referred to as S2 Fig in the main text.
(TIF)

S3 Fig Gene Ontology signatures and comparison with published heat-stress datasets. Wormcat GO enrichment analysis for genes in categories C. I **(a)** and C. II **(b),** C. III **(c)**. Each column represents GO terms related to a cluster identified in Fig 2C and 2D (e.g., “d-(1)” denotes cluster 1 from Fig 2D). Wormcat *p*-values are determined by one-side Fisher test with FDR correction. Gene and peak lists, and Wormcat outputs, are provided in S4 Data. **(d)** Venn diagrams showing overlaps between priming-induced upregulated genes (yellow) and HS-induced upregulated genes (red) identified in *glp-1(ts)* in this study, and HS-upregulated genes (gray) from published datasets by Xu and colleagues (left) and Schreiner and colleagues (right). Percentages indicate the proportion of shared HS-upregulated genes between studies. Experimental conditions are indicated below: **glp-1 (Priming):** *glp-1(ts)*, day 2 adults, 30 °C for 6 h vs. 20 °C (P1 vs. NC). **glp-1 (HS):** *glp-1(ts)*, day 2 + 12 hours adults, 35 °C for 3 h vs. 20 °C (N3.3 vs. N2). **Xu and colleagues:** N2, day 1 adults, 35 °C for 1 h vs. 20 °C. **Schreiner and colleagues:** N2, L4 larvae, 35 °C for 4 h vs. 20 °C. Referred to as S3 Fig in the main text.
(TIF)

S4 Fig No global shift in HS-induced changes in RNA expression and chromatin accessibility between the primed and naive groups in *glp-1(ts).* **(a)** Venn diagrams display the number of significantly differentially expressed genes (top panel) or peaks (bottom panel) between primed and naive groups upon a 3-h HS (light gray), and their overlap with the 6-hour HS (dark gray) in the indicated comparisons. A Fisher exact test was conducted to determine if the overlaps between the 3- and 6-h HS are statistically significant; *p*-values are displayed. **(b)** Scatter plots display genes (top panel) or peaks (bottom panel) with significant changes identified in naive or primed groups after a 6-h HS. The red line indicates the linear regression line with a 95% confidence level, and the black dashed line indicates identity line where *x* = *y*. Pearson correlation, *R*-square values, and coefficient tables are displayed. **(c)** The plot illustrates the temporal dynamics of RNA expression differences between primed and naive worms. The y-axis represents time, including time point 1 (P1 vs. N1), time point 2 (P2 vs. N2), and time point 3 (P3.6 vs. N3.6), while the x-axis represents the mean log2FC. The plot includes significant differentially expressed genes identified at any one of the three timepoints, which were grouped into eight trajectories using K-means clustering analysis. Only the mean log2FC for each trajectory is displayed as a representative trend. Trajectories of all genes in clusters 2 and 7 are displayed in **(d)**. Wormcat GO enrichment analysis for genes in clusters 2 and 7 is also shown. Wormcat *p*-values are determined by one-sided Fisher test with FDR correction. Gene lists for each cluster in (c–d) are provided in S1 Data. Venn diagrams display the number of significantly upregulated **(e)** or downregulated **(f)** genes identified at time point 1 (P1 vs. N1) and their overlap with time point 2 (P2 vs. N2) and time point 3 (P3.6 vs. N3.6) from RNA-seq analysis (Fig 3A, 3B, and 3D). Referred to as S4 Fig in the main text.
(TIF)

S5 Fig Priming-responsive changes largely restore after recovery and are distinct from Heat Shock-responsive changes in gene expression and chromatin accessibility in WT. **(a)** RNA-seq and **(b)** ATAC-seq volcano plots. Top panels: Differential gene expression or chromatin accessibility after priming (a: P1 vs. NC; b: P1 vs. N1). Red and blue indicate significantly up- or downregulated genes/peaks, respectively (adjusted *p* < 0.05, threshold shown as black horizontal line). *Bottom panels:* Differential gene expression or chromatin accessibility after recovery (P2 vs. P1), with genes/peaks colored by their P1 vs. NC or P1 vs. N1 status. Red and blue open circles represent genes/peaks previously up- or downregulated at priming, showing largely opposite regulation after recovery. Purple points indicate “newly responsive” genes/peaks that were not significantly changed in priming but became differentially regulated in P2 vs. P1. For RNA-seq **(a)**, the y-axis was truncated for visualization (Max = 45). Because NC timepoints were not collected in ATAC-seq, N1 was used as the baseline comparison. Heatmaps display genes with significant RNA expression changes **(c),** or chromatin accessibility changes **(d)** identified across the indicated comparisons, clustered by K-mean analysis using Morpheus. The colors represent normalized log2FC. The clusters in the heatmaps are arranged to parallelly present shared patterns between changes in RNA expression and chromatin accessibility. Heatmaps are classified into three categories. Category I (C. I), Priming-responsive changes: Involved clusters (1) and (2) in both (c), (d); Category II (C. I), HS-responsive changes: Involved clusters (3) and (4) in both (c), (d); Category III (C. III), Priming + HS-responsive changes: Involved clusters (5) and (6) in both **(c), (d)**. Number indicates the number of genes in the clusters (Details of the gene lists in the heatmaps can be found in S1 Data). Quality control for transcriptomic and chromatin accessibility profiles of WT: **(e)** Spearman’s correlation analysis of RNA-seq profiles for all samples, including negative control (NC), naive (N), and primed (P) groups at timepoints 1–3 across independent replicates; **(f)** Pearson’s correlation analysis of ATAC-seq profiles for all samples, including naive (N) and primed (P) groups at timepoints 1–3 across independent replicates. **(g)** Venn diagrams showing overlaps between priming-induced upregulated genes (yellow) and HS-induced upregulated genes (red) identified in WT in this study, and HS-upregulated genes (gray) from published datasets by Xu and colleagues (left) and Schreiner and colleagues (right). Percentages indicate the proportion of shared HS-upregulated genes between studies. Experimental conditions are indicated below: **WT (Priming):** N2, day 1 adults, 30 °C for 6 h vs. 20 °C (P1 vs NC). **WT (HS):** N2, day 1 + 12 h adults, 35 °C for 3 h vs. 20 °C (N3.3 vs. N2). Xu and colleagues: N2, day 1 adults, 35 °C for 1 h vs. 20 °C. Schreiner and colleague**s:** N2, L4 larvae, 35 °C for 4 h vs. 20 °C. Referred to as S5 Fig in the main text.
(TIF)

S6 Fig Differential analyses across timepoints and correlation between RNA expression and chromatin accessibility in WT. MA plots display log2FC of gene expression from RNA-seq (top panel) and chromatin accessibility from ATAC-seq (bottom panel) for the indicated comparison in naive **(a)** and primed **(b)**, and comparison between two groups **(c)**. Differential analyses were calculated using DESeq2. Significant changes (*p*-adj < 0.05) are marked in red for upregulation (log2FC > 0) and in blue for downregulation (log2FC < 0), while unchanged are marked in gray (*p*-adj ≥0.05). Numbers indicate the count of significant genes for RNA-seq data and significant peaks (and their associated genes in brackets) for ATAC-seq data for each plot. Referred to as S6 Fig in the main text.
(TIF)

S7 Fig Key regulators of heat hormesis. Thermotolerance was assessed based on the survival of WT worms treated with the indicated RNAi and after being subjected to our hormesis regimen and challenged with either 3- or 4.5-h HS. Survival curves represent combined data from multiple independent experiments (N) for *WT*_Naive or *WT*_Primed treated with empty vector (E.V.) control RNAi or *hsf-1* RNAi **(N = 3)**, *elt-2* RNAi **(N = 2),** *snpc-4* RNAi **(N = 3)**, *fos-1* RNAi **(N = 4)**, *mars-1* RNAi **(N = 3)**, and *dpy-27* RNAi **(N = 2).** The mean survival extension (in days) for each condition is indicated. Details are provided in S6 Data. Referred to as S7 Fig in the main text.
(TIF)

S8 Fig Impact of 30 °C priming on life span and reproduction. **(a)** Lifespan of *glp-1(ts)* or WT worms with or without priming was assessed at 20 °C. The figure represents combined data from four independent experiments (*N* = 4). Survival curves for *glp-1*_Naive, *glp-1*_Primed, WT_Naive, WT_Primed are shown. WT worms cultured at 25 °C from eggs to the L4 stage and then shifted to 20 °C overnight prior to priming (to match the *glp-1(ts)* culturing conditions) exhibited a smaller life span extension compared to worms continuously cultured at 20 °C (Fig 5A). **(b)** Hatching rate for each indicated time period was calculated based on the number of eggs laid and the number of hatched larvae 24 h later. Analyses were performed in WT worms with three biological replicates (*N* = 3) totaling 25 individuals. Log-rank test was used to compare mean life span in (a). Two-tailed unequal variances *t*-tests were used in (b). *** indicates *p* < 0.001. Details are provided in S1 Data. Referred to as S8 Fig in the main text.
(TIF)

S1 Data Detailed information and datasets corresponding to the indicated figures in spreadsheet format. Referred to as S1 Data in the main text.
(XLSX)

S2 Data Gene lists and log2FC values for the indicated comparisons of K-means clusters, originally identified from Morpheus (https://software.broadinstitute.org/morpheus) and further rearranged into a heatmap corresponding to Fig 2C. Referred to as S2 Data in the main text.
(XLSX)

S3 Data Peak (associated-gene) lists and log2FC values for the indicated comparisons of *K*-means clusters, originally identified from Morpheus (https://software.broadinstitute.org/morpheus) and further rearranged into a heatmap corresponding to Fig 2D. Referred to as S3 Data in the main text.
(XLSX)

S4 Data Gene ontology (GO) analysis summary for the indicated gene lists, identified using WormCat 2.0 (Category 2 and 3) [69], including statistical details, corresponding to Figs 2C, 2D and 3E, 3F. Referred to as S4 Data in the main text.
(XLSX)

S5 Data RNAi screening list in *glp-1(ts)* background, including instructions for RNAi feeding time and raw data of individual RNAi gene KD survival results. Referred to as S5 Data in the main text.
(XLSX)

S6 Data RNAi screening list in WT background, including instructions for RNAi feeding time and raw data of individual RNAi gene KD survival results. Referred to as S6 Data in the main text.
(XLSX)

S7 Data ATAC-seq analysis pipeline and instructions for execution in Linux. Referred to as S7 Data in the main text.
(TXT)

S8 Data RNA-seq analysis pipeline and instructions for execution in Linux. Referred to as S8 Data in the main text.
(TXT)

S9 Data R script for running differential expression analysis and generating an MA and Volcano plot. Referred to as S9 Data in the main text.
(R)

S10 Data R script for annotating consensus peaks. Referred to as S10 Data in the main text.
(R)

S11 Data R script for fetching promoter region sequences for motif analysis. Referred to as S11 Data in the main text.
(R)

S12 Data R script for conducting temporal dynamic analysis with time-series clustering and plotting interactive trajectories. Referred to as S12 Data in the main text.
(R)

S13 Data Matrix of raw read counts from RNA-seq data. Also available on GEO under accession number GSE312756. Referred to as S13 Data in the main text.
(TXT)

S14 Data Matrix of raw read counts in identified consensus peaks from ATAC-seq data. Also available on GEO under accession number GSE312755. Referred to as S14 Data in the main text.
(TXT)

S15 Data Reference Elements for peak annotation, adapted from a published dataset [64]. Referred to as S15 Data in the main text.
(TXT)

## Abbreviations

AP-1: activator protein-1
DCC: dosage compensation complex
GO: Gene Ontology
HS: heat shock
mtDNA: mitochondrial DNA
NPB: Nuclei Purification Buffer
QC: quality control
SNAPc: small nuclear RNA-activating complex
TAD: topologically as sociated domain
TSS: Transcription Start Site
WT: wild-type
