# Supplementary material for: Transcriptomic and chromatin accessibility profiling unveils new regulators of heat hormesis in Caenorhabditis elegans
Source: PLoS Biol. 2026 Feb 20;24(2):e3003639. doi: 10.1371/journal.pbio.3003639 (PMC12923026; doi:10.1371/journal.pbio.3003639)
Supplement: S1 Text — Referred to as S1 Text in the main text. (DOCX) [file pbio.3003639.s001.docx]

**Supplementary text**

RNA-seq and ATAC-seq data are of high quality

The data demonstrate high correlation among biological replicates for both RNA-seq and ATAC-seq results (S1A-J Fig), indicating high reproducibility of the experiments. Additionally, as expected(1), the ATAC-seq signals were enriched at regions surrounding transcriptional start sites (TSS)(2) (S1L Fig), again supporting the high quality of the data (S1 Data: RNA-seq QC, ATAC-seq QC). For RNA-seq, we detected expression of approximately 13,000 genes among the different experimental conditions. For ATAC-seq, we identified a total of 30,404 consensus peaks (S1 Data). In accordance with published analysis pipelines(3,4), we employed the narrow peak calling tool (-f BAMPE --bdg --SPMR --gsize ce -q 0.05 --call-summits) of MACS2 and the peaks were further subdivided based on their summits to achieve optimal resolution of consensus peaks. These peaks were additionally associated with 19,352 genes based on published dataset(4) (see Methods for details).

Temporal trajectories of RNA expression change between primed and naive worms

To visualize the temporal trajectories of the RNA expression differences between primed and naive worms, we conducted clustering analysis that integrated the gene expression differences between the two groups across the three timepoints. This analysis categorized the genes exhibiting significant RNA expression differences between primed and naive groups into eight distinct trajectories (S4C Fig). The distinct trajectories highlighted different degrees of RNA expression differences between primed and naive groups in response to priming and subsequent HS. Most of the trajectories (7 out of 8) followed a general pattern of peaking immediately after priming (timepoint 1) and returning to baseline by the recovery phase (timepoint 2), consistent with our earlier conclusion that the majority of the priming induced RNA expression changes are restored upon a 12-hour recovery (Fig 2A, C: C. I). Three trajectories (2, 6, 7) represented genes exhibiting differential RNA expression between primed and naive group upon a 6-hour HS (timepoint 3). Clusters 2 and 7, representing genes associated with lower RNA expression in primed compared to naive worms, are enriched for the GO terms detoxification, pathogen stress response, and lipid metabolism, aligning with our previous findings (S4D Fig). Cluster 2 captured genes that showed no significant differences between primed and naive at timepoint 1 but exhibited substantial differences at timepoint 3. On the other hand, clusters 7 and 6 captured the relatively small number of genes that showed persistently lower or higher RNA expression respectively in primed compared to naive worms across the heat hormesis regimen. Therefore, this time-series analysis led us to a similar conclusion that most priming-induced gene expression changes are transient, but also highlighted different waves of RNA expression differences between the primed and naive groups, some of which likely contribute to the stress resilience of primed worms.

We additionally compared the fold change in RNA expression or chromatin accessibility after HS in the primed vs naive worms and observed a high degree of concordance (S4B Fig), indicating that there was no global shift in HS-induced changes in RNA expression and chromatin accessibility between the primed and naive groups.

**References**

1. Yan F, Powell DR, Curtis DJ, Wong NC. From reads to insight: a hitchhiker’s guide to ATAC-seq data analysis. Genome Biol [Internet]. 2020 Feb 3 [cited 2025 Feb 16];21(1):22. Available from: https://doi.org/10.1186/s13059-020-1929-3

2. Chen RAJ, Down TA, Stempor P, Chen QB, Egelhofer TA, Hillier LW, et al. The landscape of RNA polymerase II transcription initiation in C. elegans reveals promoter and enhancer architectures. Genome Res. 2013 Aug;23(8):1339–47.

3. Daugherty AC, Yeo RW, Buenrostro JD, Greenleaf WJ, Kundaje A, Brunet A. Chromatin accessibility dynamics reveal novel functional enhancers in C. elegans. Genome Res. 2017 Dec;27(12):2096–107.

4. Jänes J, Dong Y, Schoof M, Serizay J, Appert A, Cerrato C, et al. Chromatin accessibility dynamics across C. elegans development and ageing. Lee SS, Tyler JK, editors. eLife [Internet]. 2018 Oct 26 [cited 2025 Feb 16];7:e37344. Available from: https://doi.org/10.7554/eLife.37344

5. Holdorf AD, Higgins DP, Hart AC, Boag PR, Pazour GJ, Walhout AJM, et al. WormCat: An Online Tool for Annotation and Visualization of Caenorhabditis elegans Genome-Scale Data. Genetics [Internet]. 2020 Feb 1 [cited 2025 Feb 16];214(2):279–94. Available from: https://doi.org/10.1534/genetics.119.302919
