# Supplementary figures and images for: Transcriptomic and chromatin accessibility profiling unveils new regulators of heat hormesis in Caenorhabditis elegans
Source: PLoS Biol. 2026 Feb 20;24(2):e3003639. doi: 10.1371/journal.pbio.3003639 (PMC12923026; doi:10.1371/journal.pbio.3003639)

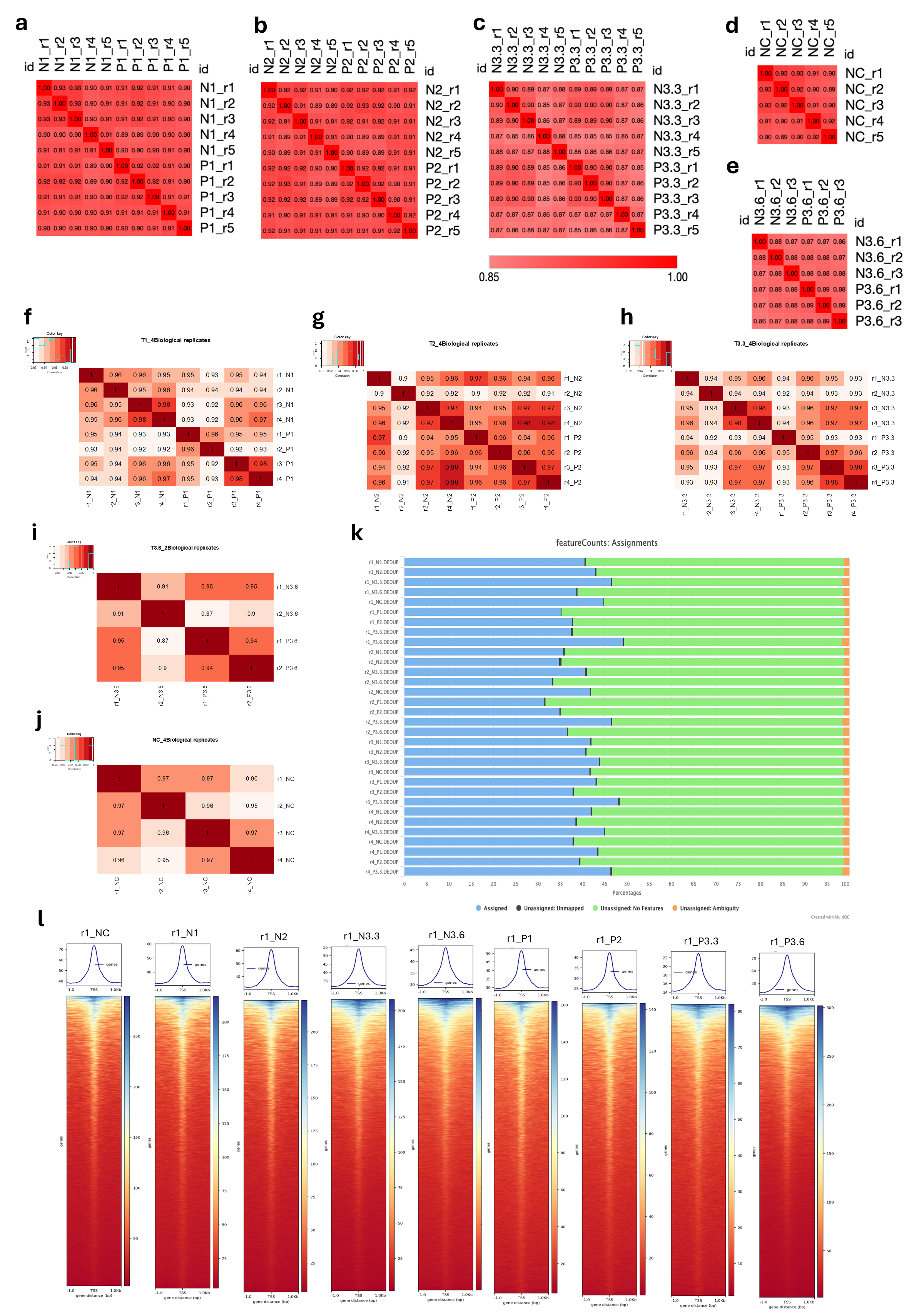

Supplement: S1 Fig — Spearman’s correlation analysis of RNA-seq profiles for both naive and primed groups of glp-1(ts) at timepoints 1 (a), 2 (b), 3.3 (c), 3.6 (e) and negative control (d) across independent replicates. Pearson’s correlation analysis of ATAC-seq profiles for both naive and primed groups at timepoints 1 (f), 2 (g), 3.3 (h), 3.6 (i), and negative control (j) across independent replicates. (k) Fraction of Reads in Peaks (FRiP) scores for individual samples, calculated using MultiQC based on featureCounts. “Assigned featureCounts” indicates mapped reads counted within identified consensus peaks (S1 Data). “Unassigned: no Features” indicates mapped reads not counted in consensus peaks. The FRiP scores within the identified consensus peaks ranged from 31% to 49% across all samples, affirming the good quality of the data. Furthermore, the FRiP scores among biological replicates were highly consistent, further supporting the reproducibility of our datasets. (l) TSS enrichment for all experimental groups in a representative biological replicate (r1): The top panel displays profile plots aggregating read coverage within 1kb upstream and downstream around TSS for all genes across the genome. The bottom panel displays heatmaps showing individual gene coverage, with each row corresponding to the TSS of a single gene, extending 1kb upstream and downstream. Colors in the heatmap indicating the level of read coverage. The plots for the remaining replicates can be found in S1 Data. Referred to as S1 Fig in the main text. (TIF) [file pbio.3003639.s002.tif]

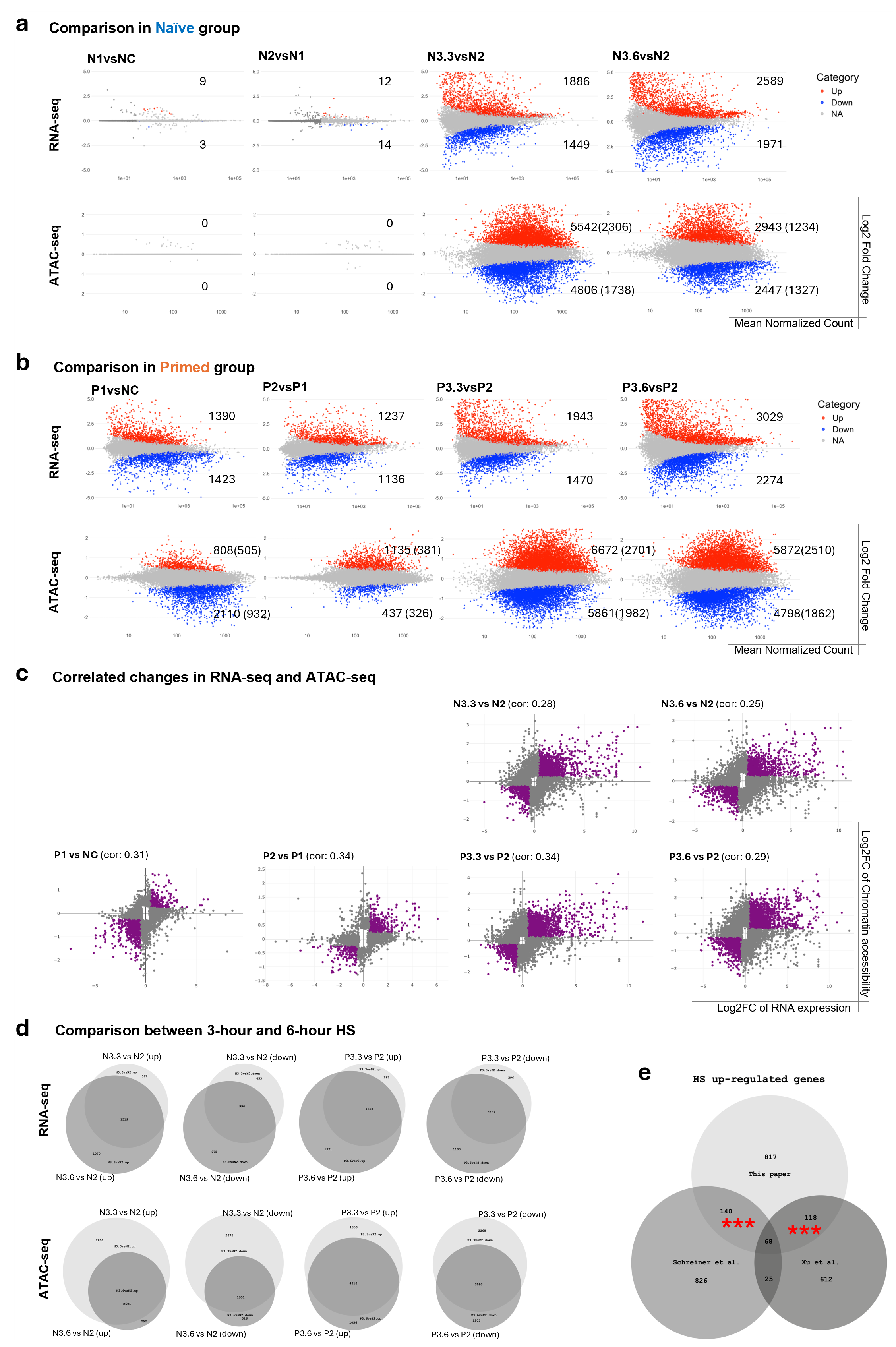

Supplement: S2 Fig — MA plots display log2FC of gene expression from RNA-seq (top panel) and chromatin accessibility from ATAC-seq (bottom panel) for the indicated comparison in naive (a) and primed (b) groups. Differential analyses were calculated using DESeq2. Significant changes (p-adj < 0.05) are marked in red for upregulation (log2FC > 0) and in blue for downregulation (log2FC < 0), while unchanged are marked in gray (p-adj >= 0.05). Numbers indicate the count of significant genes for RNA-seq data and significant peaks (and their associated genes in brackets) for ATAC-seq data for each plot. (c) Scatter plots display genes with significant changes identified in either RNA expression or chromatin accessibility for the indicated comparisons. Genes with correlated changes between RNA-seq and ATAC-seq data are highlighted in purple based on defined filter criteria: log2FC RNA expression > 0.5, <−0.5; log2FC Chromatin accessibility > 0.25, <−0.25. These genes showed upregulation in both RNA expression and Chromatin accessibility or downregulation in both RNA expression and Chromatin accessibility. Genes without correlated changes are in gray. (d) Venn diagrams display the number of significantly differentially expressed genes (top panel) or peaks (bottom panel) for the 3-hour HS (light gray) and their overlap with the 6-hour HS (dark gray) in the indicated comparison. Substantial overlaps suggest that 35 °C heat shock for 3 or 6 hours elicited many similar changes in RNA expression and chromatin accessibility. (e) Venn diagrams display the number of heat shock/stress (HS)-induced genes identified from RNA-seq, comparing our data (N3.3 vs. N2, S3A Fig) with two published datasets (Schreiner and colleagues and Xu and colleagues). Details of experimental setup and HS conditions can be found in S1 Data. Fisher exact tests were conducted to determine if the overlap between this study (This paper) and published datasets are statistically significant. *** Indicates p < 2.2e−16. Referred to as [file pbio.3003639.s003.tif]

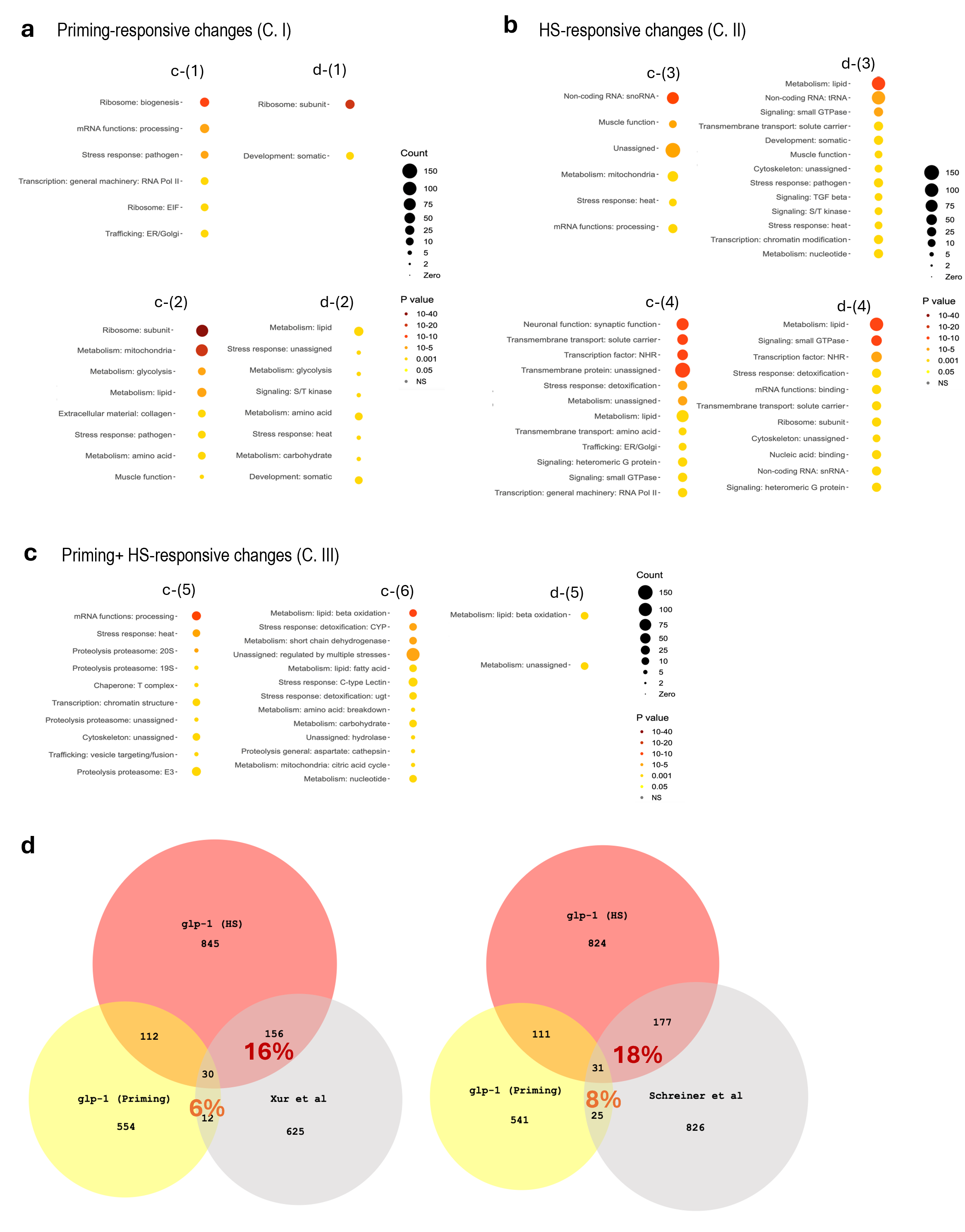

Supplement: S3 Fig — Wormcat GO enrichment analysis for genes in categories C. I (a) and C. II (b), C. III (c). Each column represents GO terms related to a cluster identified in Fig 2C and 2D (e.g., “d-(1)” denotes cluster 1 from Fig 2D). Wormcat p-values are determined by one-side Fisher test with FDR correction. Gene and peak lists, and Wormcat outputs, are provided in S4 Data. (d) Venn diagrams showing overlaps between priming-induced upregulated genes (yellow) and HS-induced upregulated genes (red) identified in glp-1(ts) in this study, and HS-upregulated genes (gray) from published datasets by Xu and colleagues (left) and Schreiner and colleagues (right). Percentages indicate the proportion of shared HS-upregulated genes between studies. Experimental conditions are indicated below: glp-1 (Priming): glp-1(ts), day 2 adults, 30 °C for 6 h vs. 20 °C (P1 vs. NC). glp-1 (HS): glp-1(ts), day 2 + 12 hours adults, 35 °C for 3 h vs. 20 °C (N3.3 vs. N2). Xu and colleagues: N2, day 1 adults, 35 °C for 1 h vs. 20 °C. Schreiner and colleagues: N2, L4 larvae, 35 °C for 4 h vs. 20 °C. Referred to as S3 Fig in the main text. (TIF) [file pbio.3003639.s004.tif]

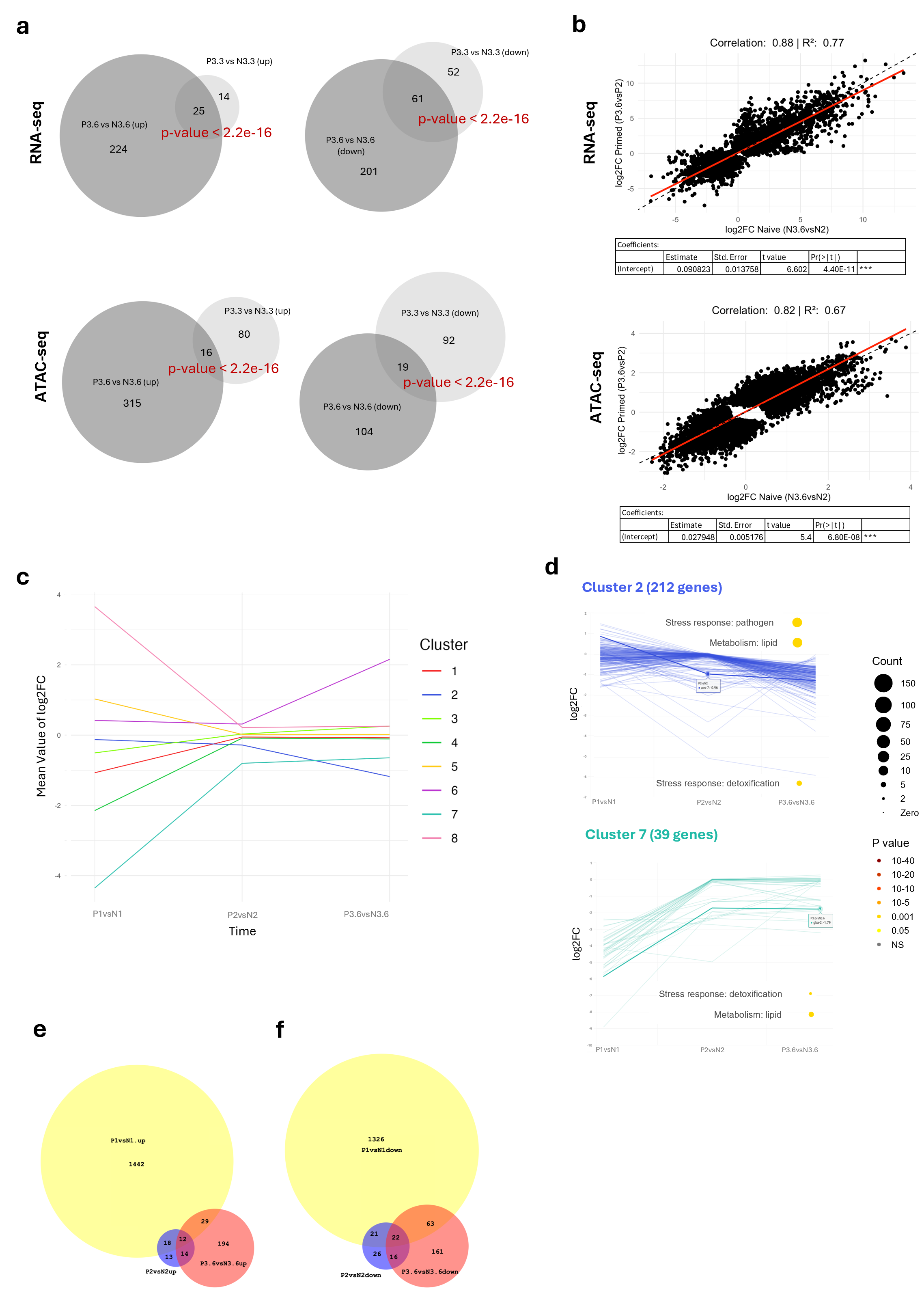

Supplement: S4 Fig — (a) Venn diagrams display the number of significantly differentially expressed genes (top panel) or peaks (bottom panel) between primed and naive groups upon a 3-h HS (light gray), and their overlap with the 6-hour HS (dark gray) in the indicated comparisons. A Fisher exact test was conducted to determine if the overlaps between the 3- and 6-h HS are statistically significant; p-values are displayed. (b) Scatter plots display genes (top panel) or peaks (bottom panel) with significant changes identified in naive or primed groups after a 6-h HS. The red line indicates the linear regression line with a 95% confidence level, and the black dashed line indicates identity line where x = y. Pearson correlation, R-square values, and coefficient tables are displayed. (c) The plot illustrates the temporal dynamics of RNA expression differences between primed and naive worms. The y-axis represents time, including time point 1 (P1 vs. N1), time point 2 (P2 vs. N2), and time point 3 (P3.6 vs. N3.6), while the x-axis represents the mean log2FC. The plot includes significant differentially expressed genes identified at any one of the three timepoints, which were grouped into eight trajectories using K-means clustering analysis. Only the mean log2FC for each trajectory is displayed as a representative trend. Trajectories of all genes in clusters 2 and 7 are displayed in (d). Wormcat GO enrichment analysis for genes in clusters 2 and 7 is also shown. Wormcat p-values are determined by one-sided Fisher test with FDR correction. Gene lists for each cluster in (c–d) are provided in S1 Data. Venn diagrams display the number of significantly upregulated (e) or downregulated (f) genes identified at time point 1 (P1 vs. N1) and their overlap with time point 2 (P2 vs. N2) and time point 3 (P3.6 vs. N3.6) from RNA-seq analysis (Fig 3A, 3B, and 3D). Referred to as S4 Fig in the main text. (TIF) [file pbio.3003639.s005.tif]

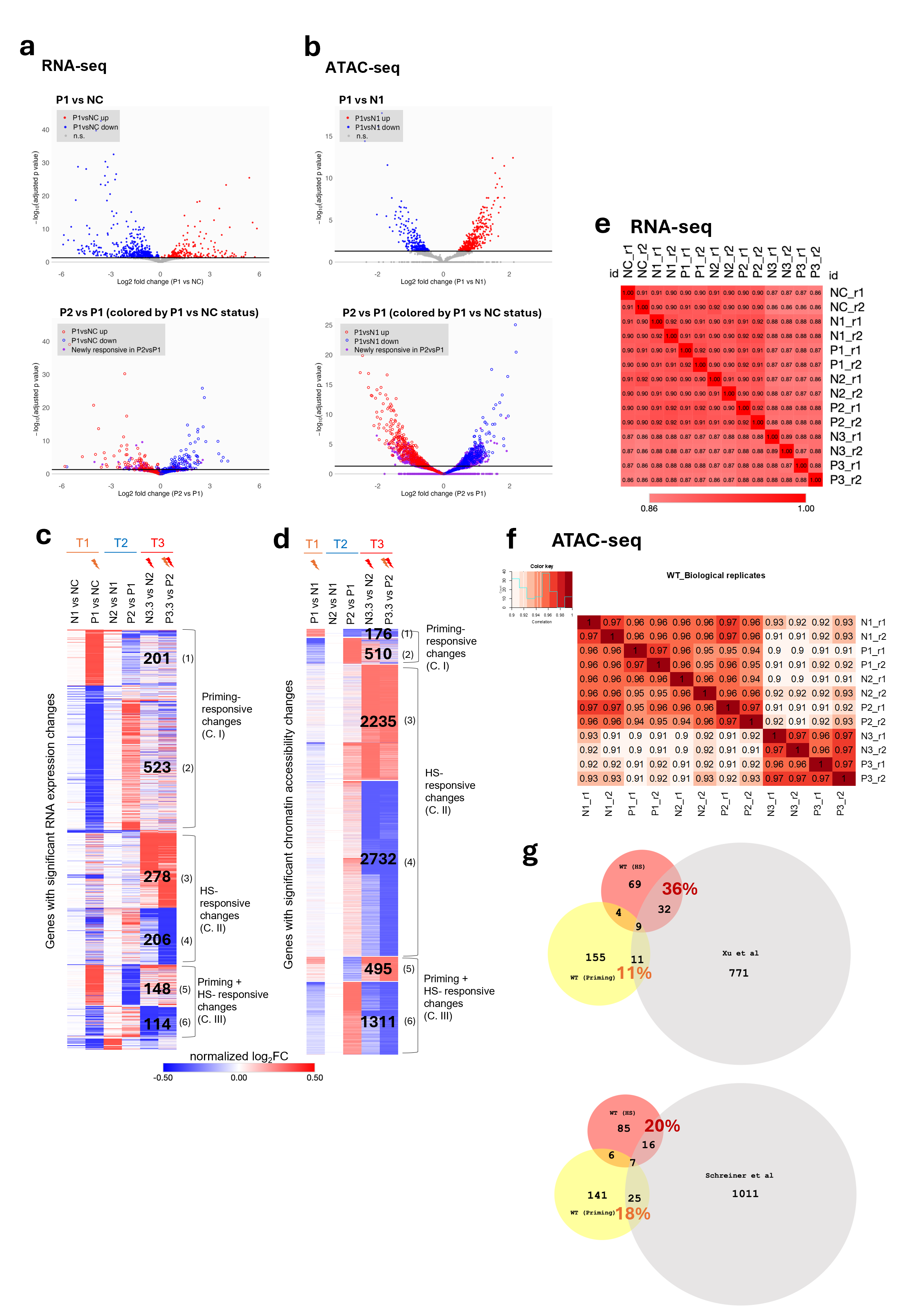

Supplement: S5 Fig — (a) RNA-seq and (b) ATAC-seq volcano plots. Top panels: Differential gene expression or chromatin accessibility after priming (a: P1 vs. NC; b: P1 vs. N1). Red and blue indicate significantly up- or downregulated genes/peaks, respectively (adjusted p < 0.05, threshold shown as black horizontal line). Bottom panels: Differential gene expression or chromatin accessibility after recovery (P2 vs. P1), with genes/peaks colored by their P1 vs. NC or P1 vs. N1 status. Red and blue open circles represent genes/peaks previously up- or downregulated at priming, showing largely opposite regulation after recovery. Purple points indicate “newly responsive” genes/peaks that were not significantly changed in priming but became differentially regulated in P2 vs. P1. For RNA-seq (a), the y-axis was truncated for visualization (Max = 45). Because NC timepoints were not collected in ATAC-seq, N1 was used as the baseline comparison. Heatmaps display genes with significant RNA expression changes (c), or chromatin accessibility changes (d) identified across the indicated comparisons, clustered by K-mean analysis using Morpheus. The colors represent normalized log2FC. The clusters in the heatmaps are arranged to parallelly present shared patterns between changes in RNA expression and chromatin accessibility. Heatmaps are classified into three categories. Category I (C. I), Priming-responsive changes: Involved clusters (1) and (2) in both (c), (d); Category II (C. I), HS-responsive changes: Involved clusters (3) and (4) in both (c), (d); Category III (C. III), Priming + HS-responsive changes: Involved clusters (5) and (6) in both (c), (d). Number indicates the number of genes in the clusters (Details of the gene lists in the heatmaps can be found in S1 Data). Quality control for transcriptomic and chromatin accessibility profiles of WT: (e) Spearman’s correlation analysis of RNA-seq profiles for all samples, including negative control (NC), naive (N), and primed (P) groups at timepoints 1– [file pbio.3003639.s006.tif]

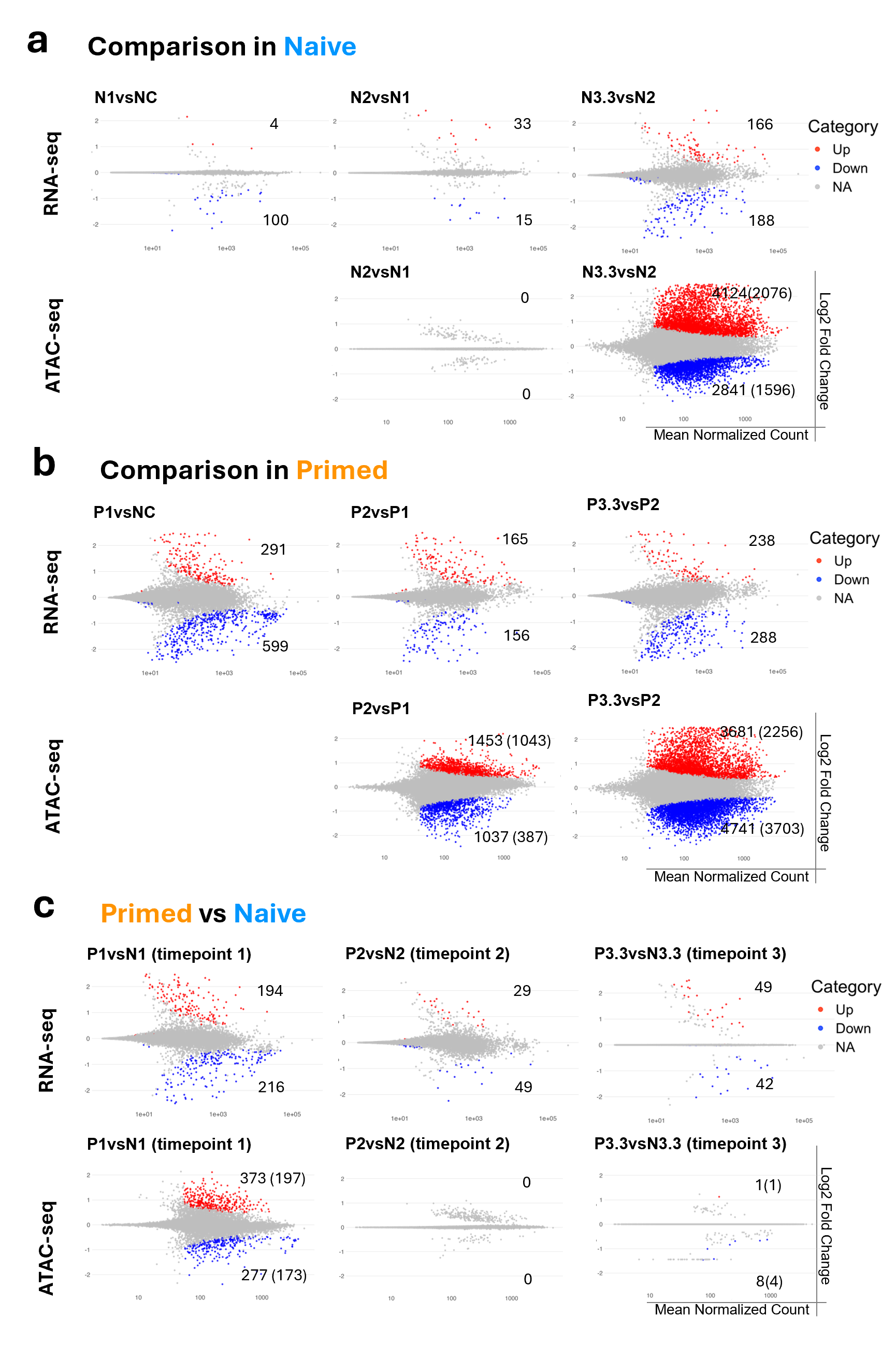

Supplement: S6 Fig — MA plots display log2FC of gene expression from RNA-seq (top panel) and chromatin accessibility from ATAC-seq (bottom panel) for the indicated comparison in naive (a) and primed (b), and comparison between two groups (c). Differential analyses were calculated using DESeq2. Significant changes (p-adj < 0.05) are marked in red for upregulation (log2FC > 0) and in blue for downregulation (log2FC < 0), while unchanged are marked in gray (p-adj ≥0.05). Numbers indicate the count of significant genes for RNA-seq data and significant peaks (and their associated genes in brackets) for ATAC-seq data for each plot. Referred to as S6 Fig in the main text. (TIF) [file pbio.3003639.s007.tif]

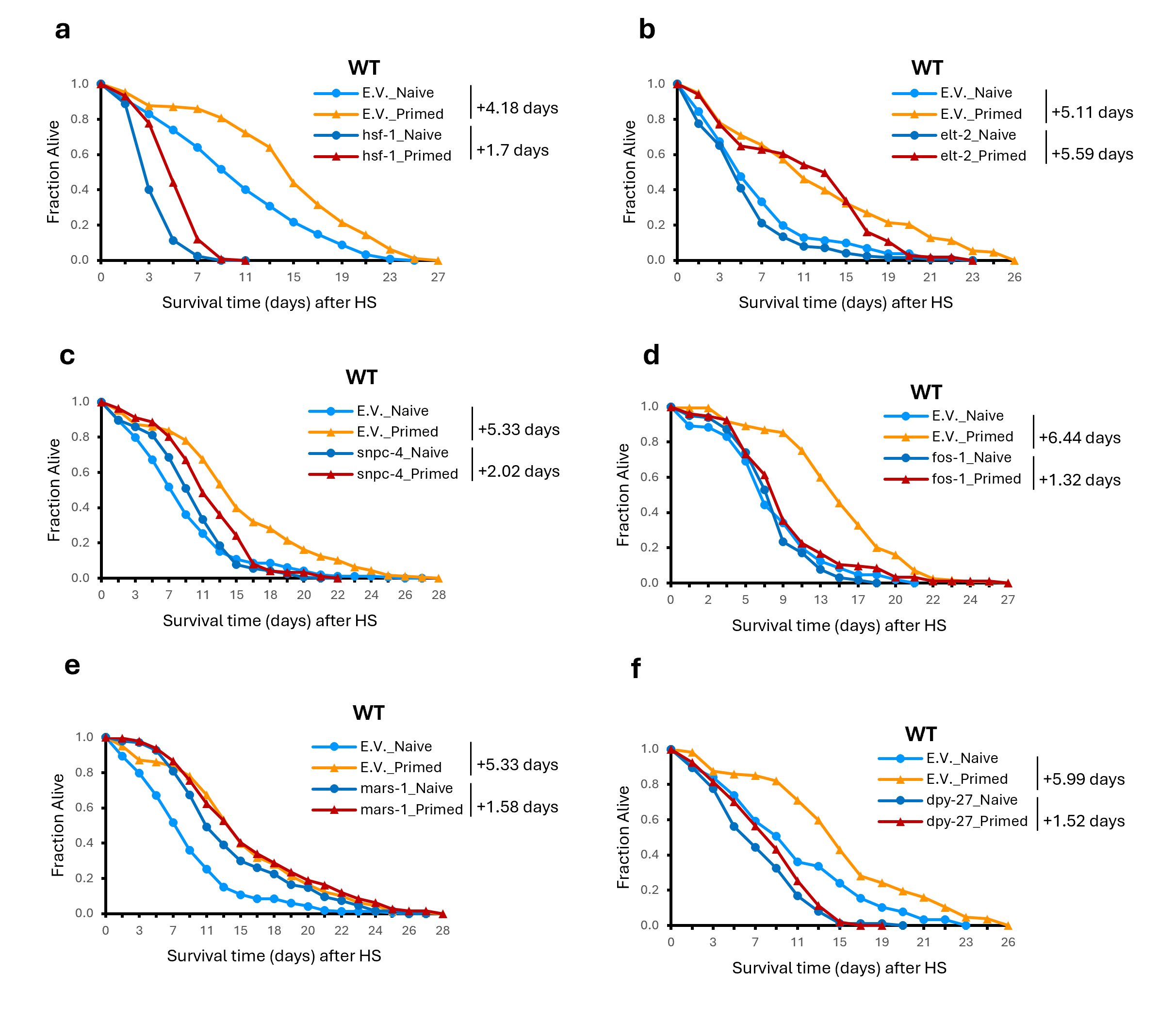

Supplement: S7 Fig — Thermotolerance was assessed based on the survival of WT worms treated with the indicated RNAi and after being subjected to our hormesis regimen and challenged with either 3- or 4.5-h HS. Survival curves represent combined data from multiple independent experiments (N) for WT_Naive or WT_Primed treated with empty vector (E.V.) control RNAi or hsf-1 RNAi (N = 3), elt-2 RNAi (N = 2), snpc-4 RNAi (N = 3), fos-1 RNAi (N = 4), mars-1 RNAi (N = 3), and dpy-27 RNAi (N = 2). The mean survival extension (in days) for each condition is indicated. Details are provided in S6 Data. Referred to as S7 Fig in the main text. (TIF) [file pbio.3003639.s008.tif]

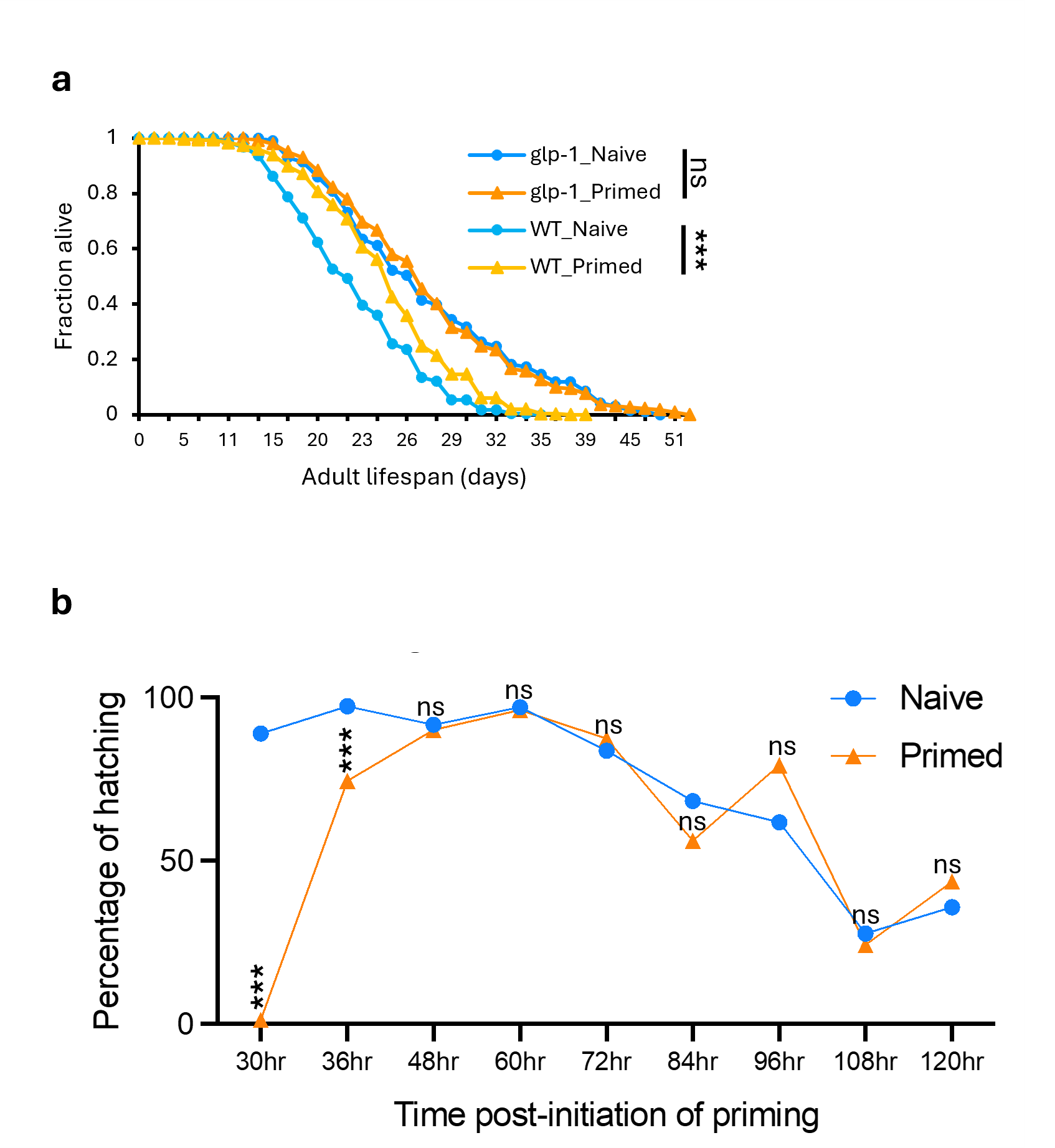

Supplement: S8 Fig — (a) Lifespan of glp-1(ts) or WT worms with or without priming was assessed at 20 °C. The figure represents combined data from four independent experiments (N = 4). Survival curves for glp-1_Naive, glp-1_Primed, WT_Naive, WT_Primed are shown. WT worms cultured at 25 °C from eggs to the L4 stage and then shifted to 20 °C overnight prior to priming (to match the glp-1(ts) culturing conditions) exhibited a smaller life span extension compared to worms continuously cultured at 20 °C (Fig 5A). (b) Hatching rate for each indicated time period was calculated based on the number of eggs laid and the number of hatched larvae 24 h later. Analyses were performed in WT worms with three biological replicates (N = 3) totaling 25 individuals. Log-rank test was used to compare mean life span in (a). Two-tailed unequal variances t-tests were used in (b). *** indicates p < 0.001. Details are provided in S1 Data. Referred to as S8 Fig in the main text. (TIF) [file pbio.3003639.s009.tif]
